# Gold nanoparticles modulate macrophage polarization to promote skeletal muscle regeneration

**DOI:** 10.1016/j.mtbio.2025.101653

**Published:** 2025-03-11

**Authors:** Lining Xu, Jiahuang Qiu, Quanzhong Ren, Dingding Wang, Anyi Guo, Ling Wang, Kedong Hou, Renxian Wang, Yajun Liu

**Affiliations:** a JST Sarcopenia Research Centre, National Center for Orthopaedics, Beijing Research Institute of Traumatology and Orthopaedics, Beijing Jishuitan Hospital, Capital Medical University, Beijing, 100035, China; b Research Center of Nano Technology and Application Engineering, School of Public Health,Dongguan Innovation Institute, Guangdong Medical University, Dongguan, 523808, China; c Department of Radiology, National Center for Orthopaedics, Beijing Jishuitan Hospital, Capital Medical University, Beijing, 100035, China; d Department of Orthopedics, Beijing Pinggu District Hospital, Beijing, 101200, China; e Department of Spine Surgery, National Center for Orthopaedics, Beijing Jishuitan Hospital, Capital Medical University, Beijing, 100035, China

**Keywords:** Gold nanoparticles, Immunomodulation, Macrophage polarization, Skeletal muscle regeneration

## Abstract

Skeletal muscle regeneration is a complex process that depends on the interplay between immune responses and muscle stem cell (MuSC) activity. Macrophages play a crucial role in this process, exhibiting distinct polarization states—M1 (pro-inflammatory) and M2 (anti-inflammatory)—that significantly affect tissue repair outcomes. Recent advancements in nanomedicine have positioned gold nanoparticles (Au NPs) as promising tools for modulating macrophage polarization and enhancing muscle regeneration. This review examines the role of Au NPs in influencing macrophage behavior, focusing on their physicochemical properties, biocompatibility, and mechanisms of action. We discuss how Au NPs can promote M2 polarization, facilitating tissue repair through modulation of cytokine production, interaction with cell surface receptors, and activation of intracellular signaling pathways. Additionally, we highlight the benefits of Au NPs on MuSC function, angiogenesis, and extracellular matrix remodeling. Despite the potential of Au NPs in skeletal muscle regeneration, challenges remain in optimizing nanoparticle design, developing targeted delivery systems, and understanding long-term effects. Future directions should focus on personalized medicine approaches and combination therapies to enhance therapeutic efficacy. Ultimately, this review emphasizes the transformative potential of Au NPs in regenerative medicine, offering hope for improved treatments for muscle injuries and diseases.

## Graphical abstract

**Figure:**
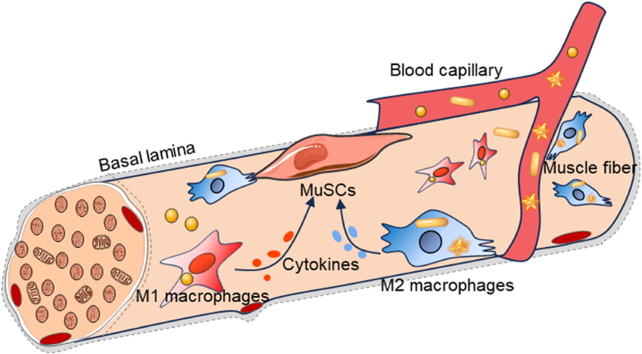

## 1 Introduction

Skeletal muscle is a highly dynamic and versatile tissue that plays a crucial role in movement, posture maintenance, and overall physical functionality. This tissue exhibits exceptional regenerative capacity, primarily attributed to the presence of muscle stem cells. Under normal physiological conditions, this regenerative ability is remarkable [1]. These resident stem cells are activated in response to muscle injury, proliferate, and differentiate to repair the damaged tissue. However, in instances of severe trauma, disease, or aging, this intrinsic regenerative process may prove inadequate, resulting in persistent muscle weakness and functional impairment [2]. The intricate interplay between the immune system and muscle regeneration is pivotal to the healing process [3,4]. Inflammation serves as a key component of the body's injury response, playing a dual role; if not properly regulated, it can lead to excessive tissue damage and impaired repair. Macrophages, as central mediators of inflammation, are critical to maintaining this balance [5]. Macrophage polarization is a dynamic process influenced by various environmental cues. The M1 phenotype, characterized by a pro-inflammatory profile, is effective during the initial stages of injury, facilitating debris clearance and combating infection. However, a subsequent shift toward the M2 phenotype, which possesses anti-inflammatory and tissue repair properties, is essential for effective regeneration [6]. Consequently, the ability to direct this polarization represents a valuable target for therapeutic intervention. Diseases associated with macrophages that impact muscle regeneration include sarcopenia, Duchenne muscular dystrophy (DMD), muscle injury, muscle ischemic disease, and exercise-induced muscle injury [[7], [8], [9]]. In these conditions, changes in macrophages are primarily reflected in their activation status and functional roles. For instance, in sarcopenia, there is an increase in TNF-α expression in macrophages, which hampers their effective conversion into the anti-inflammatory M2 phenotype, ultimately affecting the function and regeneration of muscle stem cells [10]. In the case of DMD, the pro-inflammatory state of macrophages exacerbates muscle degeneration [11]. Following muscle injury, macrophages transition from a pro-inflammatory M1 phenotype to an anti-inflammatory M2 phenotype, which facilitates the proliferation and differentiation of muscle stem cells, accelerating muscle regeneration [5]. In muscle ischemic disease, macrophages play a critical role in promoting angiogenesis and muscle regeneration by secreting various cytokines and growth factors [12]. Overall, understanding the dynamic roles of macrophages in these conditions is crucial for developing targeted therapies to improve muscle regeneration.

Gold nanoparticles (Au NPs) have garnered significant attention due to their unique physicochemical properties [13]. Their ease of synthesis, capability for functionalization with various biomolecules, and well-documented biocompatibility render them ideal candidates for biomedical applications [14,15]. The interaction of Au NPs with biological systems at the molecular and cellular levels presents an opportunity to modulate immune responses, including macrophage polarization [16]. By leveraging their ability to interact with immune cells and influence their polarization states, researchers and clinicians aim to develop strategies that can significantly enhance the healing process of skeletal muscle tissue [17]. The utilization of Au NPs to modulate macrophage polarization offers a novel approach to augment skeletal muscle regeneration [17,18]. By tailoring the properties of Au NPs, it may be possible to create an environment more conducive to tissue repair and regeneration.

This review explores the current understanding of the influence of Au NPs on macrophage behavior, elucidating the mechanisms involved and the potential therapeutic implications for muscle injuries and diseases. It outlines the essential role of macrophage polarization in muscle repair and the regulatory impact of Au NPs on these immune cells. The article highlights the promising potential of Au NPs as an innovative approach to enhancing skeletal muscle regeneration, presenting a new strategy for addressing muscle-related conditions. Additionally, it discusses the challenges that must be overcome and the research avenues that require exploration to unlock the full therapeutic potential of Au NPs in regenerative medicine. Ultimately, this review aims to provide valuable insights that could facilitate the development of more effective treatments, thereby benefiting individuals with muscular disorders.

## 2 The role of macrophage in skeletal muscle regeneration

### 2.1 Structure and composition of skeletal muscle

**Fig. 1:**
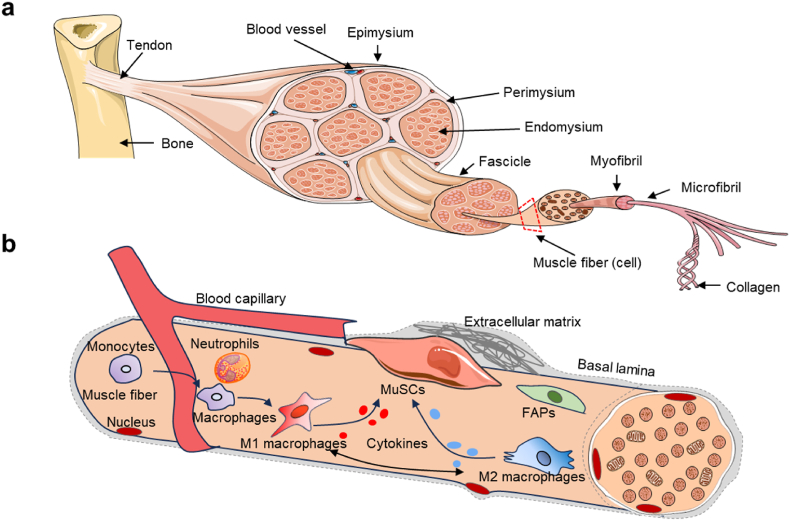
The muscular system is organized in a hierarchical manner, with the muscle stem cell (MuSC) niche playing a crucial role within this structure. (a) Illustrates the anatomical organization of muscle tissue, while (b) presents a schematic representation of the MuSC niche. MuSCs are closely associated with muscle fibers and are enveloped by the basal lamina. This close association between MuSCs and macrophages is essential for regulating the MuSC cell cycle.

Skeletal muscle, which constitutes approximately 30–40 % of an individual's body mass, is a critical tissue that requires meticulous maintenance to ensure its integrity and functionality, particularly in the context of injury, disease, or the natural aging process [19]. Skeletal muscle is a complex organ composed of various integrated tissues, beyond the muscle cells, or muscle fibers, which are organized into bundles, it encompasses blood vessels, nerve fibers, and connective tissue. Muscle fibers are classified into slow-twitch (Type I) fibers, which rely on aerobic metabolism for endurance, and fast-twitch (Type II) fibers, which are suited for high-intensity activities. The force generated by the contraction of individual muscle fibers is transmitted through various connective tissue layers, ultimately resulting in overall movement. These connective tissue layers organized into three layers of connective tissue known as mysia: the epimysium, perimysium, and endomysium (Fig. 1). The epimysium is a dense, irregular connective tissue sheath that surrounds the entire muscle, providing structural support and separating it from other tissues. Within the muscle, muscle fibers are grouped into bundles called fascicles, which are surrounded by the perimysium. Each individual muscle fiber is encased in the endomysium, a thin layer of connective tissue that helps transfer force to tendons [20]. Skeletal muscle fibers, or myofibers, are long, cylindrical cells that can be quite large, containing multiple nuclei to support their extensive protein synthesis needs. The plasma membrane of muscle fibers is called the sarcolemma, and the cytoplasm is referred to as sarcoplasm. Inside each muscle fiber, myofibrils, composed of repeating units called sarcomeres, are responsible for muscle contraction. Sarcomeres contain thick (myosin) and thin (actin) myofilaments arranged in a highly organized manner, giving skeletal muscle its striated appearance. During contraction, the myofilaments slide past one another, shortening the sarcomere and thus the muscle fiber [21]. The structure of skeletal muscle not only provides a foundation for its own contraction and movement but also offers an environment for the survival and functional expression of other cell types.

Muscle stem cells (MuSCs), also known as satellite cells, are located in the space between the basal lamina of muscle fibers and the muscle fiber membrane [22]. These cells play a crucial role in muscle growth, maintenance, and regeneration following injury. Unlike other tissue cells, muscle fibers are slender and multinucleated, formed by the differentiation and fusion of multiple MuSCs. Under normal circumstances, this property of mutual fusion is unique to MuSCs [23,24]. Consequently, the repair of muscle tissue damage relies on the fusion of MuSCs or the fusion of damaged muscle fibers. As individuals age, there is a significant decline in both the number and self-renewal capacity of MuSCs, leading to a diminished ability to repair muscle damage [25]. The satellite cell niche comprises various components, including extracellular matrix (ECM) proteins, muscle fibers, blood vessels, neuromuscular junctions, mesenchymal stem cells (such as fibro-adipogenic progenitor cells, FAPs), and tissue-resident immune cells [26]. These elements are essential for the activation of MuSCs (Fig. 1). Once activated, MuSCs enter the cell cycle, proliferate, and generate sufficient progeny that differentiate into multinucleated myotubes to regenerate damaged tissue. Throughout the muscle regeneration process, communication between satellite cells and other niche components represents a potential target for biomedical engineering aimed at enhancing the regenerative capacity of skeletal muscle tissue. In this context, nanoscale materials could provide innovative approaches for the efficient delivery of signaling molecules and drugs, thereby improving therapeutic applications.

Skeletal muscle injury triggers a characteristic inflammatory response, commencing with a rapid influx of neutrophils, followed by macrophages [27]. This inflammatory sequence aligns with the phases of muscle repair, regeneration, and growth, which include the activation and proliferation of satellite cells, ultimately leading to their terminal differentiation [28,29]. Several studies employing genetically modified animal models, specific depletion of inflammatory cell populations using antibodies, and expression profiling of inflamed muscle post-injury have illuminated the complex interplay between inflammatory cell functions and skeletal muscle injury and repair [3,12,[30], [31], [32]]. These investigations have revealed that inflammatory cells can both exacerbate injury or promote repair through interactions involving free radicals, growth factors, and chemokines. The inflammatory response to skeletal muscle injury typically induces a rapid and sequential influx of inflammatory cell populations, which can persist for days to weeks, coinciding with muscle repair, regeneration, and growth [5]. This correlation suggests a mechanistic link between inflammation and muscle regeneration, supporting the idea that muscle inflammation following exercise serves a functionally advantageous role [33]. Macrophages, in particular, exhibit a more complex role in muscle injury and remodeling compared to neutrophils, acting as abundant sources of a diverse array of growth factors, cytokines, and free radicals. Furthermore, as professional antigen-presenting cells, macrophages are crucial in modulating the cellular immune response to muscle injury, thereby influencing both the extent of damage and the subsequent remodeling processes. This complexity underscores the multifaceted nature of the inflammatory response in skeletal muscle and highlights the potential for targeted therapeutic interventions aimed at enhancing muscle recovery and regeneration.

Macrophages are a crucial component of the immune system within skeletal muscle tissue, strategically distributed throughout the muscle, particularly near blood vessels and sites of injury [27]. In healthy muscle, they maintain a baseline level of activity, continuously monitoring for signs of infection or tissue damage. Upon injury, macrophages are rapidly activated and recruited to the damaged area, where they initiate the inflammatory response. During this response, macrophages play a vital role in clearing debris and combating infection. As the inflammatory phase progresses, they transition to a state that supports tissue repair and regeneration [27]. This strategic positioning facilitates their dual role in maintaining muscle homeostasis and promoting repair. Additionally, macrophages interact with other immune cells, such as neutrophils and lymphocytes, to coordinate a comprehensive immune response [34]. This collaboration is essential for resolving inflammation and supporting muscle regeneration, highlighting the indispensable role of macrophages in skeletal muscle health.

### 2.2 Macrophage polarization in muscle skeletal muscle regeneration

Skeletal muscle regeneration is a highly complex process that depends on the dynamic interplay between immune responses and MuSC activity, with macrophages playing a pivotal role as central mediators. While other immune cells, such as neutrophils, T cells, and dendritic cells, also contribute to this process, macrophages are particularly critical due to their ability to produce a diverse array of cytokines, proteases, growth factors, soluble mediators, and ECM components. These factors collectively regulate the tissue microenvironment, orchestrating the repair process and highlighting the indispensable role of macrophages in skeletal muscle regeneration [[35], [36], [37]]. In skeletal muscle, macrophages are uniquely specialized to support muscle repair and regeneration, adapting their functions to the specific needs of this tissue. They engage in a bidirectional relationship with their surroundings, modulating the microenvironment while simultaneously being influenced by it. This interaction results in phenotypic and functional changes in response to various environmental stimuli, allowing macrophages to effectively facilitate the healing process. In contrast, macrophages in other tissues, such as the liver or lungs, fulfill additional roles—such as detoxification or respiratory defense—that are distinct from the primary focus of skeletal muscle macrophages [38,39]. This specialization highlights the critical importance of macrophages in skeletal muscle, where their activities are essential for restoring tissue integrity and function following injury.

Macrophages can be broadly classified into two polarization states: classically activated macrophages (M1) and alternatively activated macrophages (M2). M1 macrophages are induced by interferon-gamma (IFN-γ) and lipopolysaccharides (LPS), whereas M2 macrophages are further divided into subtypes: M2a (activated by IL-4 or IL-13), M2b (activated by IL-1β or LPS), and M2c (activated by IL-10, TGF-β, or glucocorticoids). However, these in vitro classifications oversimplify the complexity of in vivo conditions, where multiple stimuli coexist [40]. Recent transcriptomic and proteomic analyses have revealed a more nuanced spectrum of macrophage activation states, reflecting the intricate interplay of various stimuli [27]. Despite ongoing debates regarding macrophage polarization, this discussion adopts a simplified nomenclature that distinguishes between M1 (pro-inflammatory) and M2 (anti-inflammatory) states for clarity.

**Fig. 2:**
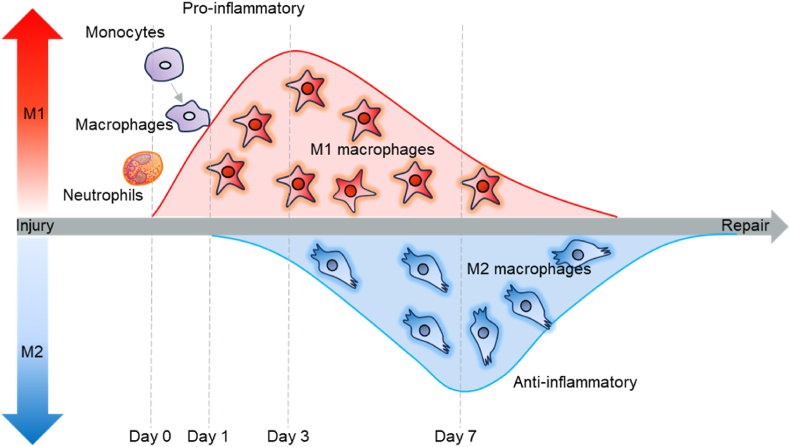
During the initial stages of inflammation, neutrophils and monocytes are the primary immune cells involved. Upon arriving at the site of injury, monocytes differentiate into macrophages, initially adopting an M1 phenotype characterized by pro-inflammatory properties essential for the early immune response. As the inflammatory process progresses, typically between days 4 and 7, a significant phenotypic shift occurs, with macrophages transitioning from the pro-inflammatory M1 state to the M2 state. The M2 phenotype is associated with anti-inflammatory and tissue repair functions, which are crucial for resolving inflammation and initiating the healing process.

M1 macrophages are characterized by their pro-inflammatory and microbicidal activities, while M2 macrophages function as anti-inflammatory cells and are often regarded as pro-resolution due to their role in producing ECM components and growth factors, such as VEGF [5]. The balance and timing of M1 and M2 macrophages are critical for effective tissue regeneration (Fig. 2). In most cases of muscle-related diseases, macrophages have an excessive M1 pro-inflammatory phenotype, so it is more important to reshape them into an M2 anti-inflammatory phenotype.

The regeneration of skeletal muscle involves the activation and differentiation of MuSCs, a process influenced by the local inflammatory environment regulated by macrophages. The regeneration of Type I fibers relies on a controlled inflammatory response, with M2 macrophages promoting tissue repair and supporting MuSC activity. In contrast, Type II fibers, particularly the fast-twitch Type IIx fibers, are more sensitive to the initial inflammatory response and require a timely transition from M1 to M2 macrophage polarization. In the context of skeletal muscle regeneration, monocytes and macrophages are recruited from the circulatory system to the site of injury. Initially, they polarize into M1 macrophages, which are pro-inflammatory and facilitate the clearance of apoptotic or necrotic muscle fibers through the release of cytokines such as IL-1β, IL-6, and TNF-α. These cytokines stimulate the proliferation of satellite cells, setting the stage for subsequent repair processes [41]. As the inflammatory phase subsides, macrophages transition to the M2 phenotype, characterized by anti-inflammatory and pro-repair properties. M2 macrophages secrete cytokines like IL-10 and TGF-β, which help suppress inflammation and promote the differentiation and fusion of myoblasts into mature muscle fibers. They also release growth factors that stimulate angiogenesis, ensuring that the regenerating muscle receives essential nutrients and oxygen. Additionally, M2 macrophages contribute to ECM remodeling, which is vital for restoring muscle structure and function [41]. Recent research by Du et al. has highlighted the pivotal role of macrophages in activating MuSCs via the release of ADAMTS1, a metalloproteinase that inhibits NOTCH1, a key regulator of MuSC quiescence, thereby triggering stem cell mobilization [42]. Moreover, growth differentiation factor 3 (GDF3), secreted by macrophages, has been identified as a crucial factor in facilitating myoblast fusion and the development of myotubes [43]. During muscle injury and aging, macrophages have been observed to secrete glutamine, which activates the mTOR signaling pathway in MuSCs [9]. This activation enhances the proliferative and differentiative capacities of MuSCs, further underscoring the multifaceted role of macrophages in muscle regeneration. Studies have also emphasized the importance of direct physical contact between macrophages and myogenic cells. In vitro studies have identified adhesion molecules such as VCAM-1, ICAM-1, PECAM-1, and the chemokine receptor CX3CR1 as key players in these interactions, which can exert anti-apoptotic effects and influence cell survival and function [44]. High-resolution imaging technologies have enabled researchers to observe these interactions in vivo. For instance, Ratnayake et al. utilized multiphoton imaging technology to show that muscle satellite cells at the site of zebrafish muscle injury must maintain continuous direct contact with specific resident macrophages until cell division is completed [27]. Conversely, He et al. developed a dual-laser multimode nonlinear light microscopy platform, demonstrating that mouse muscle satellite cells can be activated even with significantly reduced macrophage infiltration. Although their proliferation depends on macrophages, continuous contact is not necessary [45]. These findings underscore the complexity of macrophage-mediated muscle regeneration and provide new insights that could inform the development of regenerative therapies.

Macrophage polarization is a critical determinant in balancing inflammation and tissue repair during skeletal muscle regeneration. Understanding the mechanisms that drive this polarization is essential for developing strategies to enhance muscle healing. For instance, pharmacological interventions or bioactive nanoparticles could be utilized to promote the M2 phenotype, which is associated with anti-inflammatory and pro-resolution activities, while simultaneously mitigating the M1 response. Such approaches have the potential to significantly improve regenerative outcomes. A deeper understanding of the factors influencing macrophage behavior, along with the development of methods to modulate their polarization, could lead to substantial improvements in clinical outcomes for patients with muscle injuries or diseases.

### 2.3 Strategies to regulate macrophages in skeletal muscle microenvironment

Macrophages play a crucial role in muscle regeneration by influencing the proliferation and differentiation of muscle satellite cells through various signaling pathways. Their significant impact on muscle repair has positioned them as promising candidates for muscle tissue regeneration and repair therapies. Strategies for modulating macrophages within the skeletal muscle microenvironment include inhibiting monocyte/macrophage recruitment, depleting existing macrophages, and altering macrophage polarization.

(i) Inhibiting Monocyte/Macrophage Recruitment

Recruitment of monocytes/macrophages is a critical process for maintaining macrophage populations during inflammation, primarily regulated by chemokines [46]. Disrupting chemokine signaling pathways using monoclonal antibodies or small molecule inhibitors can effectively prevent the migration and differentiation of circulating monocytes/macrophages into inflammatory sites [47]. However, concerns arise regarding the potential for rapid compensation by neutrophils and the limited impact on tissue-resident macrophages [48]. Furthermore, discontinuing these inhibitors may lead to a swift release of previously sequestered monocytes/macrophages from the bone marrow, potentially triggering an excessive immune response [49]. These limitations must be carefully considered in the design of clinical trials.

(ii) Macrophage Depletion

Macrophage depletion is another strategy aimed at mitigating their negative effects during immunotherapy. Bisphosphonates, traditionally used to prevent bone metastasis or excessive bone resorption, can selectively target and kill monocytes/macrophages [17,50]. However, studies have shown that clodronate liposomes can impair muscle function recovery by depleting these cells [17,[51], [52], [53]]. Furthermore, a significant challenge with this approach is its non-specificity, as it primarily affects Kupffer cells in the liver rather than macrophages at specific inflammatory sites, which could lead to immune deficiencies.

(iii) Altering Macrophage Polarization

Changing macrophage polarization to an "immunosuppressive" phenotype can be achieved by activating pathways associated with M2 polarization or inhibiting those related to M1 polarization. For example, glabridin, a PPAR-γ agonist, can be delivered to the spleen via nanotechnology to inhibit LPS-induced M1 macrophage polarization [54]. Additionally, IL-4-modified Au NPs (IL-4-Au NPs) can promote M2 polarization following muscle injury by activating the JAK/STAT signaling pathway, thereby facilitating muscle regeneration [17].

In comparison, the inherent drawbacks of inhibiting monocyte recruitment and depleting macrophages include the loss of their essential roles as principal phagocytic and antigen-presenting cells within the immune microenvironment. While macrophages in chronic inflammation, such as that seen in aging, are continuously activated and pro-inflammatory, transforming them into an "immunosuppressive" phenotype offers the opportunity to reshape the immune-activated state. This approach could create a more favorable immune microenvironment, providing a more effective strategy for muscle regenerative immunotherapy.

## 3 Gold nanoparticles: properties, biocompatibility and macrophage-targeting

**Fig. 3:**
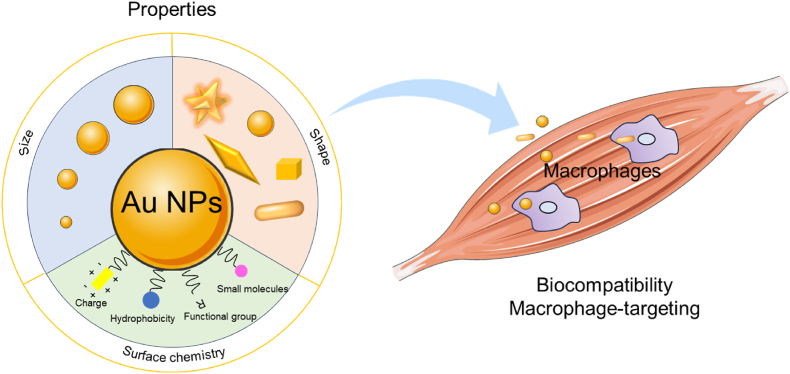
Properties of Au NPs, including size, shape, surface chemistry, as determinants of biocompatibility and targeting to muscle macrophages.

Researchers are actively exploring various approaches to restore immune equilibrium in muscles affected by disease, utilizing cytokines and other immunomodulatory substances [33,55,56]. However, many attempts have been hindered by inadequate drug metabolism and unwanted pleiotropic effects [57]. In this context, nanomedicine offers innovative solutions aimed at enhancing drug stability, improving precision targeting, and minimizing adverse effects. Au NPs have emerged as a prominent focus of research due to their significant potential in modulating the biological functions of macrophages [58]. Their unique properties make Au NPs suitable for both diagnostic and therapeutic applications in biomedicine. Key advantages of Au NPs in regulating macrophage polarization include their high specific surface area and easily tunable morphology and size, which facilitate flexible surface modifications and the coupling of various bioactive molecules, such as cytokines and nucleic acids. This capability for surface modification also allows for the attachment of different targeting ligands, enabling selective targeting of macrophages within the body. Upon introduction into the body, Au NPs are recognized and cleared by the mononuclear phagocyte system (MPS), which is rich in monocytes and macrophages [59,60]. The MPS's inherent ability to phagocytose Au NPs provides a distinct advantage for macrophage targeting. By leveraging this physiological targeting mechanism, carefully engineered Au NPs can be directed to sites of injury, where they can interact with macrophages, modulate their functions, and achieve desired immunotherapeutic effects (Fig. 3).

### 3.1 Properties of gold nanoparticles

Au NPs possess a unique combination of physical and chemical properties that make them highly valuable for various applications, particularly in medicine and materials science. One of their most notable characteristics is their size-dependent optical properties, specifically their tunable surface plasmon resonance (SPR) [61,62]. This phenomenon allows Au NPs to absorb and scatter light at specific wavelengths based on their size and shape, enabling customization for applications in imaging, drug delivery, and photothermal therapy [63,64]. In addition to their optical properties, Au NPs demonstrate excellent chemical stability and biocompatibility, which are crucial for safe use in biological environments. Their inert nature minimizes the risk of adverse reactions when introduced into the body, making them suitable candidates for various biomedical applications, including drug delivery and imaging [65]. A key advantage of Au NPs lies in their versatile surface chemistry, which allows for easy functionalization with a variety of molecules. This functionalization can enhance their effectiveness in diagnostics and targeted therapies [66]. For example, attaching antibodies or specific peptides to the surface of Au NPs enables targeted delivery to specific cell types [67]. Furthermore, Au NPs maintain good electrical conductivity, making them useful in electronic applications and biosensing technologies. Their ability to enhance fluorescence signals also plays a crucial role in various imaging applications, allowing for improved sensitivity and detection limits in biosensors [[68], [69], [70]]. This property is particularly beneficial in developing diagnostic tools where detecting low-abundance biomarkers is essential.

Overall, the versatile properties of Au NPs, combined with their potential for functionalization, position them as promising tools across a wide range of scientific and medical fields. Their unique characteristics continue to inspire innovative applications, paving the way for future advancements in nanomedicine and materials science.

### 3.2 Biocompatibility of gold nanoparticles

The biocompatibility of Au NPs, primarily due to the inert nature of gold, makes them an attractive material for various biomedical applications [71]. However, several factors can influence this biocompatibility, necessitating a deeper understanding to ensure their safe use in clinical settings [72]. One critical factor affecting the biocompatibility of Au NPs is their size. Smaller nanoparticles can penetrate cell membranes and tissues more easily, which, while beneficial for drug delivery, can also lead to increased toxicity if not properly controlled [73]. Moreover, the size of the nanoparticles impacts their circulation time in the body; smaller particles often have a longer half-life due to reduced clearance by the reticuloendothelial system. The shape of Au NPs also plays a significant role in their biocompatibility and therapeutic potential. Spherical Au NPs are generally more biocompatible than rod-shaped or star-shaped particles, which may have sharper edges and corners that could cause mechanical damage to cells and tissues [74]. Surface charge is another important factor influencing the interaction of Au NPs with biological systems. Cationic Au NPs tend to exhibit a higher potential for toxicity due to their electrostatic attraction to negatively charged cell membranes, potentially leading to membrane disruption and cellular uptake [75]. In contrast, anionic or neutral Au NPs may interact less strongly with cells, potentially reducing toxicity. Surface modifications can enhance the biocompatibility of Au NPs. For instance, the addition of polyethylene glycol (PEG) can reduce the surface charge and provide a more hydrophilic, non-fouling surface [76]. The addition of PEG to Au NPs reduces surface charge, creating a hydrophilic, non-fouling surface that minimizes toxicity and extends circulation time in the bloodstream [77]. This prolonged circulation enhances therapeutic efficacy by allowing more time for accumulation at the target site, largely due to PEGylation's ability to evade macrophage uptake [78]. By reducing non-specific interactions with immune cells, PEGylated Au NPs achieve more effective delivery. Importantly, this does not eliminate their therapeutic effects on macrophages. When functionalized with targeting ligands, PEGylated Au NPs can specifically bind to macrophage receptors at the target site, modulating macrophage behavior and promoting beneficial polarization [17,79]. Other surface modifications, such as the conjugation of targeting ligands, can improve the specificity of Au NPs for certain cell types or tissues, further enhancing their therapeutic potential [80].

Understanding the biodistribution, metabolism, clearance, and potential long-term toxicity of Au NPs is essential for assessing their safety and therapeutic efficacy. The liver and spleen, as part of the MPS, are the primary organs responsible for the clearance of nanoparticles from the bloodstream [13,81]. The kidneys also play a role, particularly for smaller particles, through filtration and excretion in urine [13,82,83]. However, prolonged accumulation in these organs could lead to toxicity, highlighting the need for careful monitoring and optimization of Au NP properties to ensure safe clearance [84,85]. Research into the long-term effects of Au NPs is crucial for understanding their safety profile. Potential concerns include changes in tissue morphology, inflammation, fibrosis, genotoxicity, carcinogenicity, and reproductive or developmental toxicity [86]. The immune system's response to Au NPs over time is also a critical area of study, as chronic inflammation could negate the therapeutic benefits of these nanoparticles in tissue regeneration. To ensure the safety of Au NPs, preclinical studies are essential. These studies involve in vitro cytotoxicity assays to evaluate potential harmful effects on cells, animal studies to assess biodistribution and pharmacokinetics, and long-term toxicity assessments to understand the effects of chronic exposure [13]. Advanced imaging techniques, such as computed tomography (CT), photoacoustic imaging (PAI), and magnetic resonance imaging (MRI), can provide valuable insights into the in vivo behavior of Au NPs, including their distribution, clearance, and potential accumulation in tissues [[87], [88], [89]]. Recent studies have shown that the metabolism and clearance of Au NPs are influenced by their size, shape, and surface chemistry. For instance, smaller Au NPs (<10 nm) are more likely to be cleared via the kidneys, while larger particles (>10 nm) are often sequestered and cleared through the liver and MPS [90,91]. The surface chemistry of Au NPs also plays a significant role in their stability and clearance. For example, PEG-coated Au NPs maintain better dispersion properties in *vivo*, facilitating their passage through the liver sinusoidal endothelium and subsequent elimination via the hepatobiliary route [92]. In contrast, Au NPs coated with chitosan (Chi) or polyethylenimine (PEI) tend to aggregate in hepatic Kupffer and endothelial cells, leading to long-term accumulation and impeding their elimination [93]. The potential long-term toxicity of Au NPs is an area of active research. While some studies have shown that Au NPs are not toxic, others have reported oxidative damage to tissues and cell lines, particularly in the liver, spleen, and kidneys. The primary toxicity and its extent are collectively determined by the characteristics, preparations, and physicochemical properties of the NPs. Functionalizing the surface of Au NPs with specific ligands can help regulate and detoxify their uptake, reducing potential toxicity [94]. Additionally, the duration and dose of exposure are important factors in determining the toxicity of Au NPs. For example, a study on the potential cerebellar toxicity of Au NPs in rats showed that both concentrations of Au NPs (25 and 50 particles per million) caused cerebellar pathology, with the toxicity being more dependent on dose than the duration of exposure [95].

Despite these challenges, the potential of Au NPs in biomedicine is vast. Their unique properties, such as tunable size, shape, and surface characteristics, make them versatile tools for a range of applications. Ongoing research focuses on optimizing the design of Au NPs to enhance their therapeutic effects while minimizing potential adverse effects [96]. This includes developing targeted delivery systems, understanding the mechanisms of cellular uptake and clearance, and establishing strategies to mitigate toxicity. In conclusion, while Au NPs offer a promising avenue for therapeutic applications in biomedicine, their safety and biodistribution require careful consideration and investigation. By addressing these challenges through rigorous preclinical studies and ongoing research, the clinical application of Au NPs can be optimized to improve healthcare outcomes.

### 3.3 Macrophage-targeting of gold nanoparticles

Macrophage-targeting with Au NPs represents a cutting-edge strategy in nanomedicine, leveraging the innate phagocytic properties of these immune cells for therapeutic benefit. As crucial components of the immune system, macrophages are renowned for their ability to engulf and digest foreign particles, including Au NPs. This natural affinity positions macrophages as ideal targets for the delivery of Au NPs in disease management [97].

The size of nanoparticles is a pivotal biophysical parameter that influences cellular targeting and uptake. In most cell types, endocytosis and pinocytosis typically mediate the internalization of nanoparticles that are around 200 nm or smaller, while phagocytosis allows for the ingestion of particles up to 10 μm in diameter [98]. This latter process is exclusive to professional antigen-presenting cells (APCs), facilitating the passive targeting of macrophages with larger particle sizes. Uptake studies have demonstrated a trend favoring the internalization of larger Au NPs by macrophages compared to their smaller counterparts [99]. Despite this preference for larger Au NPs, smaller particles are often utilized for macrophage delivery due to several considerations. For instance, the route of administration significantly influences nanoparticle behavior; intravenously administered nanomedicines must navigate the size limitations of small capillaries to avoid embolization [100,101]. Furthermore, the shape of Au NPs also impacts cellular uptake. Niikura et al. reported that macrophages exhibit the highest cellular uptake efficiency for rod-shaped Au NPs, followed by 20 nm spherical Au NPs. In contrast, 40 nm spherical Au NPs and cubic Au NPs demonstrated the lowest uptake efficiency [66]. These findings underscore the importance of both particle size and shape in the design of nanomaterials for effective macrophage targeting and therapeutic applications.

More importantly, strategic targeting of macrophages using Au NPs can be achieved through the functionalization of these nanoparticles with biomolecules that promote specific interactions with macrophages. By conjugating the surface of Au NPs with peptides, proteins, nucleic acids, or small molecules, researchers can guide the nanoparticles to engage with macrophages in a controlled manner. This targeted approach not only improves the delivery efficiency of therapeutic agents but also enhances cellular uptake, which is critical for treatment efficacy. Current targets for macrophage targeting primarily include CD44, Fcγ receptor I (FcγRI, CD64), mannose receptor (CD206), galactose-type lectin (MGL, CD301), and Toll-like receptors (TLRs).

(i) CD44

CD44 is a glycoprotein prominently expressed on macrophage surfaces, engaging with hyaluronic acid (HA) and chondroitin sulfate, which play crucial roles in mediating endocytosis [102]. The expression of CD44 varies with the polarization state of macrophages, with levels ranked as M1 > M0 ≥ M2. Notably, when CD44 expression is high (as in M1 macrophages), HA-modified nanoparticles are more effectively captured and adhered to the cell membrane. Conversely, low CD44 expression (as in M2 macrophages) favors the internalization of nanoparticles [103]. Hlaing et al. developed poly(lactic-co-glycolic acid) (PLGA) nanoparticles modified with HA (HA-PLGA-NPs) for targeted delivery of curcumin to macrophages. These modified nanoparticles exhibited increased accumulation within macrophages compared to unmodified nanoparticles [104].

(ii) CD64

Studies have identified CD64 as a promising target for macrophage targeting, particularly due to its high expression under pro-inflammatory conditions [105]. Extensive studies, including in vitro, in vivo, and ex vivo analyses of patient samples, have confirmed the elevated expression of CD64 on macrophages at chronic inflammation sites, validating its potential as a therapeutic target. Differential expression analysis of M1 and M2 macrophage surface markers using human CD64 transgenic mice and human macrophages exposed to specific inflammatory stimuli has revealed eight receptors, including CD64 and CD14, that are upregulated on M1 macrophages [106]. Notably, CD64 and CD14 were also upregulated on human peripheral blood mononuclear cell (PBMC)-derived macrophages but downregulated on M2 macrophages stimulated with IL-4. Conversely, CD206, the hemoglobin scavenger receptor CD163, and CD301 were downregulated on M1 macrophages while upregulated on M2 macrophages [106]. These findings highlight the unique characteristics of CD64, which is selectively expressed on myeloid cells and efficiently binds and internalizes monomeric immunoglobulin G (IgG). This makes CD64 an ideal target for therapeutic interventions aimed at modulating the activity of dysregulated pro-inflammatory macrophages in various pathological conditions. In vitro studies indicate that the uptake of nanoparticles is enhanced when they are conjugated with CD64 antibodies, suggesting their potential application in conditions like rheumatoid arthritis [107].

(iii) CD206 and CD301

Mannose and galactose ligands have been effectively utilized to target CD206 and CD301 on macrophages, respectively. These receptors, which are highly expressed on macrophages, have been leveraged for macrophage targeting in various disease contexts, including infectious diseases, inflammatory bowel diseases, cancers, tuberculosis, and atherosclerosis [99]. Chia et al. designed polyaniline-containing galactosylated Au NPs (PG-Au NPs) through the in *situ* polymerization of ortho-nitrophenyl-β-galactoside, assisted by gold nucleation. The high accumulation of PG-Au NPs facilitated the immune response of M2 macrophages, promoting their transition to immunogenic M1 macrophages [108]. Additionally, Ali et al. reported a positive correlation between macrophage CD206 expression and Au NP uptake, indicating that CD206 plays a significant role in enhancing Au NP internalization [109].

(iv) TLRs

TLRs are pattern recognition receptors expressed by major macrophages [110]. In a related study, Gao et al. demonstrated that cigarette smoke extract (CSE) significantly enhances the inhibitory effects of Au NP hybrids (CSE-Au NPs) on TLR4-mediated inflammatory responses. The underlying mechanism involves the adsorption of active components from CSE onto the Au NPs, which increases their cellular uptake in macrophages derived from THP-1 cells. This increased internalization not only enhances the inhibition of endosomal acidification—essential for TLR4 activation—but also promotes autophagy induction and the expression of antioxidant proteins [111]. In a separate investigation, a novel approach to TLR4 antagonism was introduced through the development of Au NPs coated with cationic glycolipids [112]. While most nano-inhibitors targeting TLRs focus on modulating TLR4 signaling, the peptide-Au NP conjugate known as P12 is notable for its broad inhibitory effects. P12 has been shown to dampen TLR4 signaling and interfere with the pathways of TLR2, TLR3, and TLR5 [113]. Subsequent research has emphasized P12's anti-inflammatory capabilities, particularly regarding gene expression triggered by LPS. This compound has been shown to reduce the production of pro-inflammatory cytokines, such as IL-12 and IFN-γ, while simultaneously promoting the expression of the anti-inflammatory IL-1Rα [114].

The targeted delivery of Au NPs to macrophages can be significantly improved by customizing their surface properties, size, and shape to optimize biodistribution and cellular interactions [115]. This precision engineering facilitates not only the efficient uptake of Au NPs by macrophages but also ensures selective targeting. By minimizing off-target effects, this approach enhances the therapeutic impact of the nanoparticles, making them more effective in treating various conditions [72].

In addition, the delivery methods of Au NPs also play a crucial role in both localized and systemic muscle-targeted therapies, directly influencing the precision, efficiency, and safety of treatment. Localized delivery methods, such as intramuscular injection or hydrogel-based carriers, allow Au NPs to be efficiently concentrated at the site of muscle injury, effectively reducing inflammation and accelerating tissue repair [116,117]. This approach not only significantly enhances therapeutic outcomes but also minimizes systemic side effects, making it suitable for treating localized muscle injuries or chronic muscle disorders. In contrast, systemic delivery methods, such as intravenous injection, are more appropriate for systemic muscle diseases or injuries involving multiple sites [116]. Through circulation, Au NPs can be widely distributed to muscle tissues throughout the body and preferentially accumulate in specific areas of injury, enabling comprehensive treatment. Furthermore, Au NPs can be combined with other therapeutic strategies, such as stem cell therapy, physical rehabilitation, or drug delivery, to achieve synergistic effects and further enhance muscle regeneration and functional recovery [118,119]. These diverse delivery methods and application scenarios highlight the immense potential of Au NPs in muscle injury repair and disease treatment.

## 4 Influencing factors and mechanisms of gold nanoparticles in modulating macrophage polarization

The regulation of macrophage polarization by Au NPs is a complex process shaped by the physical and chemical properties of the nanoparticles as well as their interactions with macrophage biology. A thorough understanding of these influencing factors and underlying mechanisms is crucial for developing targeted therapies and nanomaterials with tailored properties for immunomodulatory applications. Particularly in the context of muscle regeneration, this knowledge can play a pivotal role in enhancing therapeutic strategies aimed at promoting effective macrophage behavior. By modulating macrophage polarization, Au NPs can potentially influence the inflammatory environment, thereby facilitating muscle repair and regeneration in various disease states. Ultimately, harnessing these mechanisms can lead to improved therapeutic outcomes in muscle regeneration and other related conditions.

### 4.1 The factors of gold nanoparticles in regulating macrophage polarization

(i) Size Effect

The impact of Au NPs of varying sizes on macrophage polarization is a complex and multifaceted process with significant implications for immunotherapy and tissue repair. Wang et al. compared Au NPs with diameters of 10 nm, 20 nm, and 50 nm, finding that 50 nm Au NPs were optimal for promoting M2 macrophage polarization while inhibiting the secretion of inflammatory factors [120]. Compared to smaller Au NPs (10 nm and 20 nm), larger Au NPs (50 nm) can induce M2 macrophage polarization by inhibiting the NF-κB signaling pathway, which subsequently inhibits p65 protein activation and nuclear translocation. Additionally, it was confirmed that 50 nm Au NPs enhance macrophage phagocytosis of *Staphylococcus aureus* (*S. aureus*) by overexpressing TREM2 and promoting the interaction of VAMP8 with STX17 and SNAP29, thereby enhancing the fusion of autophagosome lysosomes and facilitating the degradation of intracellular bacteria. Moreover, these larger Au NPs promote vascular differentiation and bone formation, thereby accelerating tissue regeneration at the site of infection [120]. Gao et al. also discovered that larger Au NPs (20 nm) exhibited a stronger inhibitory effect on the activation of TLR4 and its downstream cytokine production (CCL2, CCL4) in THP-1 cell-derived macrophages compared to smaller Au NPs (5 nm and 13 nm). This enhanced inhibitory effect may be attributed to the higher cellular uptake and stronger endosomal pH buffering capacity of 20 nm Au NPs [121].

Conversely, Zhang et al. found that smaller-sized Au NPs (5 nm and 20 nm) were more readily endocytosed by macrophages than larger-sized Au NPs (50 nm and 100 nm), demonstrating a more significant effect on macrophage polarization. In particular, 20 nm PEG-modified Au NPs (PEG-Au NPs) exhibited the ability to inhibit M2 macrophage polarization in vitro, thereby enhancing the efficacy of immunotherapy. This may be due to the smaller size of 5 nm and 20 nm Au NPs, which allows for more effective penetration of the cell membrane, increased endocytosis by macrophages, alkalinization of lysosomes, and enhanced membrane permeability. Consequently, this induces inhibition of autophagic flow, resulting in impaired lysosomal function, inhibition of M2 polarization, and activation of M1-type pro-inflammatory responses through the NF-κB signaling pathway [122]. Su et al. further demonstrated that smaller PG-Au NPs (polyaniline-based glyco structure modified Au NPs) were more effective in inducing the transition from M2-type to M1-type macrophages. Specifically, the smaller the size of PG-Au NPs, the stronger the effect on inducing M1-type macrophage polarization. The size-dependent effects observed in their experiments indicated that PG-Au NPs at 32.2 nm and 29.8 nm had comparable effects, while those at 26.4 nm and 18.3 nm exhibited stronger effects. The size order was as follows: 18.3 nm < 26.4 nm < 29.8 nm < 32.2 nm. This may be attributed to the increased uptake of smaller PG-Au NPs by cells via endocytosis (such as macropinocytosis and clathrin-mediated endocytosis), which leads to a more pronounced endoplasmic reticulum (ER) stress response. ER stress activates the tyrosine kinase SYK, which in turn regulates downstream signaling pathways, including NF-κB and STAT6, thereby increasing the secretion of cytokines that promote inflammatory responses (such as CXCL10/IP-10) while decreasing the secretion of immunosuppressive cytokines (such as IL-10) [123]. Cheng et al. also found that smaller Au NPs (4 nm) were more effective than larger Au NPs (14 nm) in promoting the polarization of macrophages towards the M1-type inflammatory phenotype and inhibiting the M2-type anti-inflammatory phenotype. This effect is attributed to the greater uptake of smaller Au NPs by macrophages, resulting in lysosomal damage, cytoplasmic vacuolation, and pyroptosis, which subsequently enhances the immune response, promotes M1-type macrophage polarization, and activates the secretion of pro-inflammatory cytokines through the NF-κB signaling pathway [124]. In addition, studies have indicated that the size of Au NPs affects their binding to fibrinogen. Smaller Au NPs are more likely to bind to fibrinogen, thereby interacting with the giant integrin receptor Mac-1 on the macrophage membrane surface, which activates the NF-κB signaling pathway and leads to the release of inflammatory cytokines [125]. This highlights the intricate relationship between the size of Au NPs, their interactions with macrophages, and the subsequent immune response. However, it is essential to recognize that size alone cannot fully account for the observed polarization behavior. This indicates that additional factors, such as shape, surface ligands and functionalization, also significantly influence the polarization state.

(ii) Shape Effect

The shape of Au NPs, such as spherical-shaped Au NPs (Au SNPs), rod-shaped Au NPs (Au NRs), and star-shaped Au NPs (Au TNPs), also plays a pivotal role in determining their interactions with macrophages and influencing their polarization status [58]. High aspect ratio (AR) Au NRs have been shown to penetrate macrophages more efficiently, yet they also exit more rapidly. This dynamic behavior may facilitate their long-term retention within the microenvironment and effective excretion via the liver [126]. Such interactions are crucial for the biodistribution and clearance of Au NPs within the body. Research conducted by Kang et al. further elucidates the impact of anisotropic ligand nanogeometry on macrophage adhesion and polarization. By utilizing Au NRs with varying aspect ratio, they demonstrated that Au NRs with high anisotropy (AR4 and AR7) promote macrophage interaction through integrin β1, in contrast to Au NRs with low anisotropy (AR1 and AR2). The highly anisotropic Au NRs enhance macrophage adhesion, promote the development of actin filaments and vinculin, and facilitate the M2 polarization of macrophages via the ROCK molecular pathway, while inhibiting the M1 state [127]. In contrast, Xia et al. investigated the differential responses of RAW 264.7 macrophages to Au SNPs and Au TNPs. They found that Au SNPs induced a rounded, amoeba-like morphology in macrophages, accompanied by increased surface detachment, suggesting a potential activation state. However, this morphological change was not associated with a significant production of pro-inflammatory molecules such as TNF-α and reactive oxygen species (ROS), indicating a weaker inflammatory response. Conversely, Au TNPs, with their multi-branched structure, were linked to cell enlargement and the production of TNF-α and ROS, indicative of a more pronounced activation of macrophages and a stronger inflammatory response [128]. Bhoge et al. reported that, compared to Au SNPs and Au TNPs, Au NRs, despite being rapidly internalized by macrophages, elicited a poor immunogenic response. In contrast, Au TNPs demonstrated strong secretion of pro-inflammatory cytokines, suggesting a greater efficacy in inducing macrophage polarization. This difference may be attributed to the localization of Au NRs primarily within the mitochondrial region, whereas Au TNPs exhibit a richer localization in the late endosome and lysosome regions. This localization facilitates the degradation and release of ligand molecules, which interact with TLR7/8 and TLR9 receptors in endosomes, thereby activating immune responses [129].

(iii) Surface Chemistry Effect

The surface chemical properties of Au NPs, including surface modifications and functionalization, significantly influence macrophage polarization, which is crucial for immune responses and tissue regeneration. Numerous studies have demonstrated that factors such as surface charge, ligand density, and the presence of specific functional groups on Au NPs can modulate the proportion and activation state of macrophages [123,130,131]. For instance, it has been shown that 11-mercaptoundecanoic acid (MUA)-modified Au NPs can reduce LPS-induced macrophage infiltration by approximately 11 %, increase the percentage of M2 macrophages threefold, and decrease the proportion of M1 macrophages by 59 % [132]. Cao et al. found that CpG-modified Au NPs (CpG-Au NPs), which act as an agonist of TLR9, enhanced intracellular uptake efficiency and in vivo stability of CpG. These modifications facilitated the repolarization of M2-type macrophages to M1-type, significantly promoting the expression of M1-type macrophage markers (e.g., iNOS and IL-12) while inhibiting the expression of M2-type markers (e.g., CD206 and Ym1) [133]. Furthermore, Pallares et al. reported that Au NPs with low ligand densities (approximately 3 CpGs per Au NP) exhibited poor cellular uptake, resulting in negligible immune activation and cytokine release. In contrast, when ligand density increased to approximately 55 CpGs per Au NP, the immune stimulation was enhanced, maximizing the release of pro-inflammatory cytokines and chemokines such as TNF-α, RANTES, and MIP-2. This indicates that ligand density is a critical factor influencing the immunogenicity of Au NPs by enhancing cellular uptake [134]. Moreover, Au NPs with specific ligand spacing and sizes can induce M2 macrophage polarization, which is associated with anti-inflammatory and pro-regenerative properties. For example, Kim et al. demonstrated that ligands with a 3 nm spacing promoted the binding of integrins across adjacent ligands, thereby facilitating integrin aggregation. This high-density integrin aggregation contributes to the adhesion formation of macrophages, promoting M2-type polarization. However, increasing the ligand spacing from 3 nm to 17 nm significantly hinders macrophage adhesion, leading to inflammatory M1-type polarization. This finding suggests that ligand spacing plays a dominant role in regulating integrin binding and macrophage polarization [135]. Additionally, it was observed that increasing ligand size from 7 nm to 20 nm only slightly enhanced macrophage adhesion, while no significant effect was noted for ligands with a size of 13 nm. This implies that ligand size has a minor impact on macrophage polarization; however, a size threshold of approximately 20 nm may promote integrin aggregation and macrophage adhesion [135]. It has also been found that conjugates of Au NPs with peptides can be recognized by mouse bone marrow macrophages, whereas peptides or nanoparticles alone do not elicit this response. Further studies indicate that macrophage activation is more closely related to the arrangement pattern of peptides on the surface of Au NPs than to the length and polarity of the peptides. Different peptide sequences can lead to varying biochemical reactions, which correlate with the degree of order in the peptide coating [136]. The surface charge of Au NPs also influences macrophage interactions. Positively charged Au NPs are more readily internalized by cells, forming aggregates with intracellular proteins and potentially delaying exocytosis. In contrast, PEG modified Au NPs migrate individually within the cytoplasm and are quickly excreted, thereby reducing the likelihood of aggregation and inflammation [58]. Furthermore, the combination of Chi with Au NPs has been shown to reduce M1 polarization and enhance M2 polarization in macrophages, which is beneficial for tissue repair [137]. Similarly, Au NPs modified with specific peptides or proteins, such as ovalbumin (OVA) [131], can activate M1 polarization in macrophages. Additionally, numerous studies have reported that upon entering the organism, Au NPs rapidly adsorb protein molecules to form a "protein corona", which can significantly affect the interaction and polarization between Au NPs and macrophages [[138], [139], [140]]. Chen et al. highlighted the temporal changes in the protein corona components on the surface of Au NPs and their subsequent effects on macrophage polarization. They found that pristine Au NPs induced a macrophage M2 phenotype when incubated with serum for 4 h. However, as the incubation time extended to 12 h, the proportion of immune proteins in the protein corona increased, leading to the induction of a macrophage M1 phenotype. This study underscores the importance of the protein corona's composition and its dynamic nature in influencing immune responses, emphasizing the need for further investigation into the mechanisms underlying these phenomena [141].

**Fig. 4:**
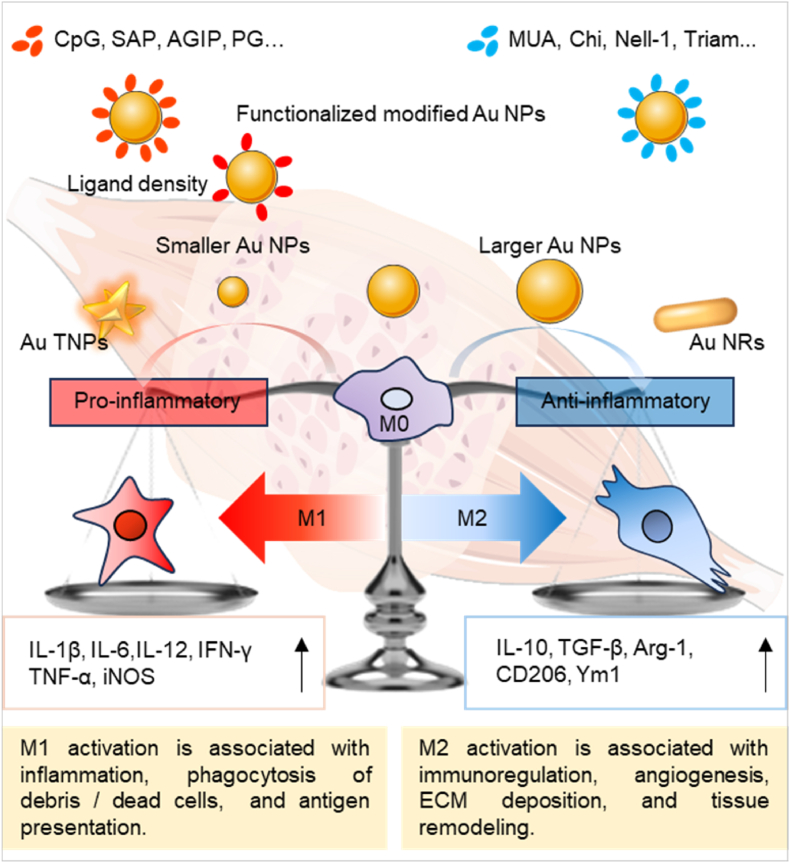
Au NPs with different physicochemical properties and functionalization are used to regulate the polarization phenotype of macrophages and release pro- or anti-inflammatory cytokines, which play a key role in tissue damage and repair and regeneration.

In summary, the size, shape, and surface chemistry of Au NPs significantly influence their interactions with macrophages and the resulting polarization states, which hold critical implications for immunotherapy and tissue repair (Fig. 4). Typically, larger Au NPs tend to promote M2 polarization, which is beneficial for anti-inflammatory responses and tissue regeneration. In contrast, smaller Au NPs often induce M1 polarization, thereby enhancing immune responses. Similarly, the shape of Au NPs, such as rod-shaped versus star-shaped, affects their cellular uptake and the polarization of macrophages, with high aspect ratio nanoparticles promoting M2 polarization and multi-branched structures eliciting stronger inflammatory responses. Surface modifications, including ligand density and charge, further modulate macrophage interactions and polarization, highlighting the importance of tailored nanoparticle design for specific therapeutic outcomes. However, a significant limitation in current research is the predominant focus on single-dimensional physicochemical properties, which has led to conflicting findings and limited generalizability across different studies. This narrow approach often overlooks the complex interplay of multiple factors that contribute to the structure-activity relationship of Au NPs, necessitating a more holistic examination of their effects to better understand and optimize their therapeutic potential in clinical applications. Future investigations should aim to integrate these multifaceted aspects to elucidate the underlying mechanisms governing macrophage polarization and enhance the efficacy of Au NP-based therapies.

### 4.2 Mechanisms of gold nanoparticles in modulating macrophage polarization

The ability of Au NPs to influence macrophage polarization is multifaceted, involving various mechanisms such as the regulation of cytokine production, interaction with cell surface receptors, activation of intracellular signaling pathways, modulation of cellular redox balance, metabolic regulation, and epigenetic modifications. In this section, we summarize the key mechanisms through which Au NPs modulate macrophage polarization, highlighting their complex roles in immune response modulation.

(i) Modulation of Cytokine Production

The modulation of cytokine production by Au NPs represents a critical mechanism by which these nanoparticles can influence macrophage polarization. Research has demonstrated that Au NPs can alter the cytokine profile of macrophages, promoting either an M1 or M2 phenotype depending on the specific context and application. The ability of Au NPs to stimulate the production of anti-inflammatory cytokines, such as IL-10 and TGF-β, while simultaneously reducing the secretion of pro-inflammatory cytokines like IL-6, TNF-α, and IL-1β, underscores their potential in immunomodulation and regenerative medicine. Below, we discuss several major macrophage cytokines and the potential regulatory role of Au NPs on their expression:

IL-10 is a potent anti-inflammatory cytokine that suppresses the pro-inflammatory activities of macrophages and other immune cells. It inhibits the production of TNF-α, IL-1β, and other cytokines, thereby reducing inflammation and promoting tissue repair. Au NPs have been shown to upregulate IL-10 expression, potentially shifting the macrophage phenotype towards the M2 phenotype, which is associated with anti-inflammatory and reparative functions [16,132,142].

TGF-β plays a multifaceted role in immune regulation and tissue repair. It contributes to the resolution of inflammation and promotes extracellular matrix production, which is essential for wound healing. Au NPs can enhance TGF-β production, thereby facilitating the transition of macrophages to a phenotype that supports tissue regeneration and fibrosis resolution [16,132].

IL-6 is a multifunctional cytokine that plays a critical role in the acute phase response, inflammation, and immune regulation. It is produced by various cell types, including macrophages, in response to inflammatory stimuli and can act in an autocrine or paracrine manner to influence the behavior of neighboring cells. IL-6 production is often induced through the activation of inflammatory signaling pathways, such as NF-κB, JNK, STAT, and Nrf2 pathways. Au NPs can interfere with these pathways, potentially by affecting the phosphorylation and nuclear translocation of key transcription factors, thereby reducing the transcription of IL-6 genes [16,143,144].

TNF-α is a primary cytokine involved in systemic inflammation and is a key driver of the M1 macrophage phenotype. Elevated levels of TNF-α are associated with chronic inflammation and tissue damage. Au NPs can reduce TNF-α secretion, which may help mitigate excessive inflammation and promote a more regulated immune response [16,143,145].

IL-1β is another pro-inflammatory cytokine that contributes to the initiation and amplification of inflammatory responses. Au NPs can decrease IL-1β levels, which may be particularly beneficial in conditions where unchecked inflammation can lead to severe tissue damage [145].

Indeed, Au NPs possess the unique ability to not only steer macrophages towards an anti-inflammatory M2 phenotype but also promote a pro-inflammatory M1 polarization by enhancing or attenuating the expression of specific cytokines. This dual-regulatory capacity offers flexibility in the application of Au NPs for the treatment of various diseases. For instance, Pallares et al. found that CpG-Au NPs significantly increased the release of TNF-α, RANTES, and MIP-2 from macrophages [134]. Similarly, Guan et al. reported that G-quadruplex-modified Au NPs-treated macrophages exhibited increased release of pro-inflammatory cytokines and chemokines, such as IL-6, MCP-1, RANTES, and TNF-α, which can function as immune adjuvants [146].

(ii) Interaction with Cell Surface Receptors

The interaction of Au NPs with cell surface receptors on macrophages is a critical aspect of their potential application in modulating immune responses and facilitating tissue regeneration.

IL-4 alpha receptor (IL-4Rα) is a unique member of the common-gamma chain (γc) family of receptors [147,148]. When IL-4 binds to this receptor, it activates the JAK-STAT signaling pathway, especially STAT6, leading to a polarization transition of macrophages from a pro-inflammatory M1 type to a M2 type with anti-inflammatory and tissue repair functions, thus playing a key role in regulating immune responses and promoting tissue regeneration [149]. Raimondo et al. utilized IL-4-Au NPs for injection into the skeletal muscle of damaged mice to investigate their effects on muscle tissue [17]. The study revealed that IL-4-Au NPs can upregulate the expression of CD206, with the induction of M2a-type macrophages by IL-4-Au NPs being both higher and more stable than that induced by soluble IL-4. Furthermore, mice treated with IL-4-Au NPs exhibited accelerated clearance of CD45^+^ immune cells following injury, alongside an increase in the proportion of M2a-type macrophages and a decrease in M1-type macrophages. Notably, these mice demonstrated significant improvements in muscle contractility and speed compared to the control group, indicating that IL-4-Au NPs promote muscle fiber regeneration and functional recovery by inducing M2a-type macrophage polarization [17]. Furthermore, IL-4-Au NPs were utilized in studies involving muscular dystrophy to enhance muscle fiber area, contractility, and speed [79]. These findings suggest that Au NPs can serve as a promising immunomodulatory therapy, offering potential benefits for patients with various muscle diseases characterized by inflammation. This underscores the significance of in vivo studies in advancing our understanding of effective therapeutic strategies.

TLRs constitute a family of pattern recognition receptors that are essential for the innate immune system to detect pathogen-associated molecular patterns (PAMPs) and danger-associated molecular patterns (DAMPs) [150]. TLRs are expressed on the surface of macrophages and other immune cells, and their activation leads to the production of pro-inflammatory cytokines, chemokines, and the initiation of adaptive immune responses [121,133,134,142,151,152]. The interaction of Au NPs with TLRs can modulate the immune response in various ways. For instance, when Au NPs engage with TLRs, they can mimic the presence of pathogens and trigger a signaling cascade that activates macrophages. This activation can promote M1 polarization, characterized by a pro-inflammatory phenotype that leads to muscle inflammation, potentially resulting in harmful effects [153]. Conversely, specific modifications to the surface of Au NPs can inhibit TLR activation, potentially fostering an anti-inflammatory environment that favors M2 macrophage polarization. M2 macrophages play a crucial role in tissue repair, and wound healing by suppressing the immune response and promoting angiogenesis [142,150,152]. The ability of Au NPs to interact with TLRs and influence macrophage polarization is highly dependent on their physical and chemical properties, including size, shape, surface charge, and surface coating. For example, Bastús et al. synthesized Au NPs conjugated with amyloid growth inhibitory peptide (AGIP) and sweet arrow peptide (SAP), resulting in AGIP-Au NPs and SAP-Au NPs. Their study revealed that macrophages did not recognize AGIP, SAP, or Au NPs individually; rather, only the conjugates were capable of activating TLR4 and inducing the production of pro-inflammatory cytokines [154]. In addition, Gao et al. demonstrated that Au NPs inhibit the TLR4 signaling pathway by regulating the endosomal acidification process. They found that 20 nm Au NPs exhibit more effective TLR4 inhibition compared to 13 nm Au NPs, attributed to their higher cellular uptake rate and stronger endosomal pH buffering capacity [121]. Additionally, Ali et al. reported that Au NPs synthesized using honey (Honey-Au NPs) can downregulate LPS-induced IL-6 secretion by macrophages through the inhibition of the TLR4/NF-κB pathway [155].

(iii) Activation of Intracellular Signaling Pathways

Intracellular signaling pathways, such as NF-κB, MAPKs, JAK/STAT, and Nrf2, play a central role in regulating inflammatory responses and determining the polarization state of macrophages [16,144,156].

NF-κB is a key transcription factor that controls the expression of multiple inflammation-related genes. In macrophages, the activation of NF-κB is often associated with M1-type polarization and promotes the production of pro-inflammatory cytokines [157]. In the case of 50 nm Au NPs, it has been shown that they promote M2 macrophage polarization by inhibiting the NF-κB pathway [120]. This inhibition involves preventing the activation and nuclear translocation of the p65 subunit of NF-κB, which is crucial for the expression of pro-inflammatory genes. Conversely, Au NPs have demonstrated the ability to inhibit NF-κB activation by suppressing Akt activity, which subsequently reduces the expression of IFN-β and the phosphorylation of STAT1. This suggests that Au NPs can modulate macrophage polarization by blocking the activation of both NF-κB and STAT1 pathways, leading to a decrease in the production of nitric oxide (NO) and inducible nitric oxide synthase (iNOS) [158]. Furthermore, peptide-conjugated Au NPs (Peptide-Au NPs) have exhibited stronger anti-inflammatory activity by reducing the activation of NF-κB and interferon regulatory factor (IRF), as well as the secretion of CCL2 and CCL4 cytokines in THP-1-derived macrophages [121]. Au NPs have also been found to suppress NF-κB activation induced by high glucose levels, which is critical for the regulation of inflammation and the immune response.

The MAPK family, which includes ERK1/2, JNK, and p38, is involved in the regulation of various physiological processes such as cell response, proliferation, and apoptosis [159,160]. Au NPs have been reported to suppress the secretion of inflammatory cytokines by inhibiting the MAPK pathway, thereby reducing the overall inflammatory response [120,143,144]. By inhibiting the ERK1/2 MAPK/Akt/mTOR signaling pathway, Au NPs reduce the activation of NF-κB and the expression of inflammatory genes [156]. Additionally, Nell-1-conjugated Au NPs (Nell-1-Au NPs) may exert their effects on macrophage polarization by regulating both the MAPKs and Wnt/β-catenin signaling pathways [143].

The JAK/STAT pathway plays a central role in cytokine signaling and is involved in cell differentiation, development, and inflammatory responses [161,162]. The balance between STAT1 activation and STAT3 and STAT6 activation is critical for fine-tuning macrophage polarization and function. When STAT1 activation is dominant, it causes M1 macrophage polarization. In contrast, dominant activation of STAT3 and STAT6 leads to M2 macrophage polarization [163]. Liao et al. also found that in addition to MAPKs and the Wnt/β-catenin pathway, Nell-1-Au NPs induce M2 polarization in macrophages by regulating JAK1, JAK3, STAT1, and STAT3 expression, thereby inhibiting hyperinflammation by regulating the ratio of M2/M1 macrophages [143].

Moreover, Au NPs have been shown to promote the differentiation of M2 macrophages and facilitate the reprogramming of macrophages from the M1 to the M2 phenotype by activating the Nrf2 pathway, an antioxidant pathway distinct from the traditional STAT6 pathway [164]. Au NPs can activate or modulate these pathways, promoting the expression of genes associated with either the M1 or M2 macrophage phenotype [164]. This ability to influence transcriptional regulation is a critical aspect of how Au NPs direct macrophage polarization. Luo et al. further assessed the activity of dendrimer-encapsulated Au NPs with three surface modifications—primary amine (NH_2_-Au NPs), hydroxyl (OH-Au NPs), and quaternary ammonium ions (CH_3_-Au NPs)—in regulating macrophage function and antioxidant responses through the Nrf2-dependent pathway [165]. The study found that NH_2_-Au NPs and CH_3_-Au NPs, but not OH-Au NPs, significantly activated the Nrf2-antioxidant response element pathway in THP-1 cells. Among the three, NH_2_-Au NPs considerably increased mRNA levels and antioxidant activities of heme oxygenase 1 and NAD(P)H quinone dehydrogenase 1 in THP-1 cells. Furthermore, Nrf2 activation by NH2-Au NPs enhanced the phagocytic ability of THP-1 macrophages [165].

(iv) Regulation of Cellular Redox Balance

The regulation of cellular redox balance is a critical aspect of cellular homeostasis and function, particularly in the context of immune cell function and macrophage polarization. Reactive oxygen/nitrogen species (ROS/RNS), such as superoxide anions (O_2_^−^), hydrogen peroxide (H_2_O_2_), hydroxyl radicals (OH), and peroxynitrite anion (ONOO-) are byproducts of cellular metabolism and can act as signaling molecules or cause oxidative stress, depending on their concentration and the cellular environment. Au NPs have been shown to modulate the redox balance within cells, which can have significant implications for macrophage behavior and polarization [166]. By influencing the levels of ROS/RNS, Au NPs can indirectly affect the expression of cytokines and other immune mediators, which are key factors in the polarization process of macrophages.

In the case of M1 macrophage polarization, an increase in ROS production is typically associated with the pro-inflammatory response. ROS can activate various signaling pathways that lead to the production of pro-inflammatory cytokines, such as TNF-α, IL-1β, and IL-6 [167]. These cytokines play a crucial role in pathogen clearance and the initiation of adaptive immune responses. Therefore, Au NPs that enhance ROS levels could potentially promote M1 polarization, enhancing the immune response [168]. Xia et al. compared Au SNPs and Au TNPs with two different shapes of Au NPs and found that compared with Au SNPs, the uptake of multibranched Au TNPs did not cause significant changes in macrophage morphology or adhesion, but was associated with ROS production and promoted macrophage activation [128].

Conversely, M2 macrophage polarization is characterized by an anti-inflammatory and pro-healing phenotype. In this context, Au NPs that reduce ROS levels can favor M2 polarization by inhibiting the expression of pro-inflammatory cytokines and promoting the production of anti-inflammatory cytokines, such as IL-10, which can suppress the immune response and facilitate tissue repair [144]. Park et al. found that Au NPs have the ability to decompose H_2_O_2_, but have a limited effect on intracellular ROS levels. Triam-Au NPs modified by triaminol (Triam) functionalization can be effectively taken up by M1 macrophages through caveolae and clathrin-mediated endocytosis, and repolarize M1 to M2 macrophages. However, triaminol alone can only reduce proinflammatory responses and cannot promote repolarization [169]. In addition, some studies have shown that treatment with Au NPs is able to increase the activities of superoxide dismutase (SOD), catalase (CAT) and glutathione peroxidase (GPx), which are important intracellular antioxidant defense systems, thereby affecting the generation of ROS [170]. Rizwan et al. found that hyperglycemia triggers the activation of signaling pathways related to the pathogenesis of atherosclerosis by increasing the production of intracellular ROS/RNS [156]. Au NPs are able to reduce oxidative stress caused by hyperglycemia, restore intracellular antioxidant levels, and reduce the accumulation of reactive oxygen/nitrogen species in cells. Studies have also shown that Au NPs can also reduce the levels of NO and iNOS by regulating the transcription of iNOS genes [145].

(v) Metabolic Regulation

In addition to the previously discussed mechanisms, Au NPs can also modulate macrophage polarization through the regulation of cellular metabolic pathways. Metabolism plays a crucial role in immune cell function, including macrophage polarization [171,172]. The switch between glycolysis and oxidative phosphorylation (OXPHOS) can influence the activation state and the functional outcome of macrophages [173]. Au NPs can impact the metabolic reprogramming of macrophages, which in turn affects their polarization. For instance, M1 macrophages tend to exhibit a more glycolytic metabolism, which supports their pro-inflammatory activities, while M2 macrophages may rely more on oxidative phosphorylation, aligning with their anti-inflammatory and tissue repair functions. A recent investigation has illuminated the subtle yet significant effects of Au NPs on the metabolic activity and polarization state of macrophages, which meticulously examined the influence of Au NPs on bone marrow-derived dendritic cells (BMDCs) and bone marrow-derived macrophages (BMDMs) from mice. It was discovered that while Au NPs did not modulate the basal mitochondrial respiration of BMDCs, they notably enhanced the basal respiration of BMDMs, especially when these cells were subjected to IL-4 stimulation. This enhancement was further characterized by an increased ATP yield in BMDMs, implying that Au NPs may stimulate metabolic vigor in these cells by elevating their energy requirements. Moreover, the research revealed that Au NPs have the potential to shape the polarization trajectory of BMDMs through the modulation of their metabolic profiles, the metabolic perturbations induced by Au NPs could gently steer BMDMs towards a polarization state akin to M2 macrophages [174]. The interaction of Au NPs with cells can lead to changes in the expression and activity of metabolic enzymes, transporters, and transcription factors that regulate metabolic pathways [[175], [176], [177]]. By doing so, Au NPs can influence the availability of metabolic substrates and the generation of metabolic intermediates that serve as signaling molecules or regulators of gene expression. This metabolic reprogramming can subsequently affect the production of cytokines and other effector molecules, thereby modulating the polarization process. Moreover, Au NPs can potentially alter the macrophage's ability to respond to metabolic stress or to utilize specific nutrients, which can have a significant impact on the macrophage's functional phenotype. The ability of Au NPs to regulate macrophage metabolism provides an additional dimension to their immunomodulatory capabilities and offers novel avenues for therapeutic interventions. Understanding the interplay between Au NPs and macrophage metabolism is essential for developing strategies to manipulate macrophage polarization for therapeutic purposes. Further research is needed to elucidate the precise metabolic pathways affected by Au NPs and how these changes translate into functional outcomes in macrophage biology and immunology.

(vi) Epigenetic Regulation

Au NPs may also exert their effects through epigenetic mechanisms, which involve modifications to the DNA and associated proteins that do not change the DNA sequence itself but can alter gene expression [178]. By influencing DNA methylation and histone modifications, Au NPs can affect the accessibility of cytokine genes to the transcriptional machinery, thereby influencing the polarization of macrophages [171]. This adds another layer of complexity and precision to the regulatory effects of Au NPs on macrophage function.

**Fig. 5:**
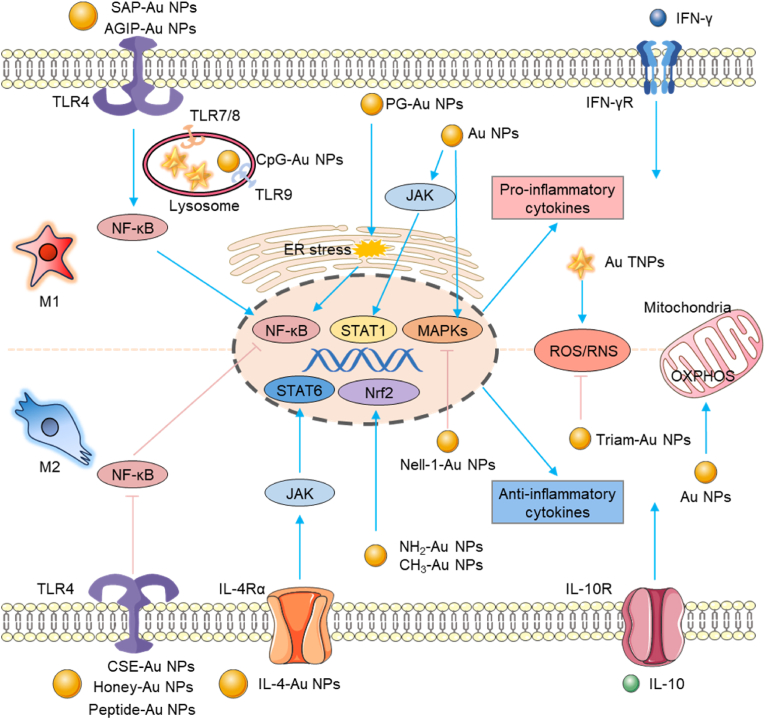
Mechanisms of Au NPs regulating macrophage polarization involve regulation of cytokine production, interaction with cell surface receptors, activation of intracellular signaling pathways, regulation of cellular redox balance, metabolic regulation, and epigenetic regulation.

In summary, the modulation of macrophage polarization by Au NPs is a complex and multifactorial process that involves various mechanisms, including cytokine production, interactions with cell surface receptors, activation of intracellular signaling pathways, regulation of cellular redox balance, metabolic reprogramming, and epigenetic modifications (Fig. 5). These intricate mechanisms collectively contribute to the fine-tuning of macrophage responses, underscoring the potential of Au NPs as versatile therapeutic agents for modulating immune function and promoting tissue regeneration. However, current research is often limited by a focus on isolated aspects of these mechanisms, neglecting the complex interactions and feedback loops that exist among them. This narrow approach can lead to conflicting findings and reduce the generalizability of results, thereby hindering the development of comprehensive therapeutic strategies. Additionally, variability in experimental conditions—such as nanoparticle size, shape, surface chemistry, and specific macrophage environments—complicates the understanding of true structure-activity relationships. To advance the field, future research should adopt a more integrative perspective that examines the interplay between these various mechanisms and their cumulative effects on macrophage polarization. This holistic understanding is essential for optimizing Au NP design and enhancing their therapeutic efficacy in clinical settings, ultimately paving the way for more effective interventions in immune modulation and inflammation.

## 5 Other applications of gold nanoparticles in skeletal muscle regeneration

**Fig. 6:**
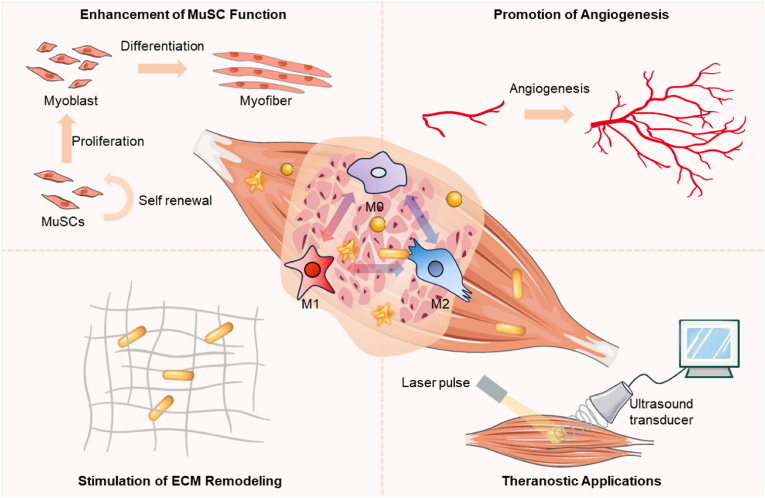
Applications of Au NPs in skeletal muscle regeneration. In addition to regulating macrophage polarization and affecting skeletal muscle regeneration, Au NPs can also promote skeletal muscle repair by directly enhancing muscle stem cell function, promoting angiogenesis, stimulating ECM remodeling, and play an active role in muscle tissue imaging and cell tracking.

In addition to regulating macrophage polarization and influencing skeletal muscle regeneration, Au NPs can also facilitate skeletal muscle repair by directly promoting angiogenesis, stimulating ECM remodeling, and enhancing the function of MuSCs. Furthermore, Au NPs play an active role in muscle tissue imaging and cell tracking (Fig. 6).

(i) Enhancement of Muscle Stem Cell Function

MuSCs are essential for muscle regeneration, and stimulus-responsive Au NPs present a promising approach to enhance this process due to their unique physicochemical properties. These nanoparticles play a crucial role in promoting the proliferation, differentiation, and functionality of MuSCs by directly activating key signaling pathways vital for muscle cell growth and repair. This activation ultimately facilitates the formation of new muscle fibers, which are necessary for restoring muscle function and strength. By integrating stimulus-responsive Au NPs into tissue engineering constructs, advanced scaffolds can be developed that dynamically interact with the cellular environment. These scaffolds not only provide mechanical support but also deliver targeted biochemical cues that further stimulate muscle regeneration. This innovative approach holds great potential for improving therapeutic strategies aimed at enhancing muscle recovery and overall functionality [179,180]. This enhancement occurs by activating signaling pathways that are crucial for muscle cell growth and repair. Consequently, Au NPs promote the formation of new muscle fibers, which are essential for the recovery of muscle function and strength. Research has demonstrated that mechanical stimulation drives myoblast differentiation. Ramey-Ward et al. developed a gel complex containing Au NRs that can deform up to 5 μm when triggered by near-infrared (NIR) light pulses. The results indicate that light-triggered hydrogel mechanical deformation enhances myogenesis in C2C12 myoblasts, primarily through the activation of the ERK signaling pathway, myocyte fusion, and myosin expression [181]. Additionally, the authors found that stimulation of the Au NR-containing gel complex in proximity to the nucleus resulted in the localization of the transcriptional activator YAP-1 to the nucleus, further underscoring the role of YAP-1 in the mechanotransduction of C2C12 cells [182].

Beyond mechanical stimulation, mild thermal stimulation of muscle cells presents an intriguing approach for regulating their function. Marino et al. utilized photothermal conversion to remotely stimulate striated muscle cells by employing gold nanoshells (Au NSs) in conjunction with NIR radiation, demonstrating effectiveness in inducing myotube contraction [183]. Furthermore, Ge et al. investigated the effects of Au NPs and gold-silver nanoparticles (Au-Ag NPs) on the proliferation, myogenic differentiation, and associated molecular mechanisms of C2C12 cells, as well as their impact on skeletal muscle tissue regeneration in *vivo* [184]. The study found that both Au NPs and Au-Ag NPs promoted myoblast attachment and proliferation with minimal cytotoxicity. Under various incubation conditions, including normal and differentiation media, these nanoparticles significantly enhanced myogenic differentiation by upregulating the expression of myosin heavy chain (MHC) proteins and key myogenic genes such as MyoD, MyoG, and Tnnt-1. Further analysis revealed that Au NPs and Au-Ag NPs activated the p38α mitogen-activated protein kinase (p38α MAPK) signaling pathway, which is crucial for enhancing myogenic differentiation. Importantly, in vivo studies conducted using a rat tibialis anterior muscle defect model demonstrated that both Au NPs and Au-Ag NPs significantly promoted skeletal muscle regeneration [184]. These findings underscore the potential of Au NPs in directly influencing muscle stem cell function and tissue repair, highlighting their promise for clinical translation in the treatment of muscle injuries and disorders.

(ii) Promotion of Angiogenesis

Angiogenesis plays a pivotal role in supplying oxygen and nutrients to regenerating muscle tissue, and Au NPs have been demonstrated to enhance this process [185]. By loading or stimulating the secretion of proangiogenic factors such as VEGF, Au NPs facilitate the migration, proliferation, and tube formation of endothelial cells, leading to the development of new blood vessels [186]. This enhanced vascular network not only supports regenerating muscle tissue but also improves the integration of the regenerated tissue with the surrounding muscle, which is crucial for functional recovery [187]. VEGF-A165 is known to stimulate the proliferation and migration of vascular endothelial cells as well as the formation of tubular structures, which are key steps in the angiogenesis process. Wei et al. found that Au NPs exhibited pro-angiogenic activity comparable to that of free VEGF-A165 by promoting the proliferation and migration of human umbilical vein endothelial cells (HUVECs), as well as the formation of tubular structures. Furthermore, the functionalization of VEGF-A165 on the surface of Au NPs increased its local concentration, thereby enhancing its efficiency in promoting angiogenesis. This approach also facilitated the sustained release of VEGF-A165, providing continuous angiogenic stimulation throughout the regeneration process [188].

(iii) Stimulation of ECM Remodeling

The ECM is a dynamic network that provides essential structural support and biochemical cues necessary for muscle regeneration [189]. Au NPs can significantly influence ECM remodeling by regulating the activity of enzymes such as the matrix metalloproteinase (MMP) family [190]. This regulation is critical for maintaining the balance between the degradation and synthesis of ECM components, which is vital for effective muscle regeneration. By modulating the ECM, Au NPs can create a more favorable environment for muscle cell attachment, migration, and differentiation. For instance, MMP-1 plays a crucial role in ECM degradation, and gallic acid-modified Au NPs (GA-Au NPs) have been shown to possess a strong ability to inhibit high-glucose-mediated MMP-1-induced ECM degradation. This inhibition helps preserve the integrity of the ECM, thereby supporting the regenerative processes of muscle cells and enhancing overall muscle recovery. Through these mechanisms, Au NPs contribute to a more conducive microenvironment for muscle regeneration [191].

(iv) Theranostic Applications

Theranostics, which combines therapy and diagnostics, is an emerging field where Au NPs show great promise in skeletal muscle regeneration. By functionalizing Au NPs with imaging agents, researchers can monitor the progress of regeneration in real-time, providing valuable insights into the regenerative process [[192], [193], [194]]. Additionally, these nanoparticles can be conjugated with therapeutic agents that promote muscle repair, allowing for targeted intervention and treatment. This dual functionality of Au NPs in theranostics offers a novel approach to regenerative medicine, with the potential to improve clinical outcomes and patient care [195]. For example, Jokerst et al. utilized Au NRs as contrast agents for PAI and quantified mesenchymal stem cells in muscle tissue of rodents. The low background of this technique allows for in vivo imaging of up to 100,000 cells, offering significant advantages over traditional cell imaging technologies such as PET and MRI [195].

Au NPs have demonstrated significant potential in the field of skeletal muscle regeneration. They not only regulate macrophage polarization, which influences skeletal muscle regeneration, but also directly enhance the functionality of MuSCs. Furthermore, Au NPs promote angiogenesis, stimulate ECM remodeling, and play an active role in muscle tissue imaging and cell tracking. Specifically, Au NPs enhance the proliferation and differentiation of MuSCs by activating key signaling pathways, facilitating the formation of new muscle fibers. Concurrently, they promote angiogenesis by either loading or stimulating the secretion of angiogenic factors, thereby providing essential oxygen and nutrients for regenerated muscle tissues. Additionally, Au NPs influence ECM remodeling by modulating the activity of MMP family enzymes, which creates a favorable environment for the attachment, migration, and differentiation of muscle cells. In terms of diagnostic and therapeutic applications, Au NPs serve as imaging agents that enable real-time monitoring of the regeneration process. When combined with therapeutic agents, they facilitate targeted intervention and treatment, thereby offering novel strategies to enhance clinical outcomes in skeletal muscle regeneration and improve patient care.

## 6 Challenges and future directions

The use of Au NPs as a tool to modulate macrophage polarization for enhancing skeletal muscle regeneration presents a promising avenue of research. However, several challenges and areas for future investigation need to be addressed to fully realize the potential of these nanoparticles in clinical applications. Through continued research and optimization, these challenges can be tackled, paving the way for the development of safe and effective Au NP-based therapies for muscle injuries and diseases.

(i) Optimization of Nanoparticle Design

Optimizing the design of Au NPs is crucial for improving their biocompatibility, therapeutic efficacy, and long-term effectiveness in biomedical applications. Key parameters, including size, shape, surface charge, and surface ligands, significantly influence their interactions with biological systems. For instance, smaller nanoparticles may enhance cellular uptake, while larger ones may be more suitable for targeted applications. The surface charge plays a vital role in determining interactions with cell membranes, impacting both uptake and immune response. To promote sustained efficacy, researchers should explore various surface modifications, such as coating Au NPs with biocompatible polymers like PEG, which can create hydrophilic, non-fouling surfaces that minimize recognition by phagocytic cells and extend circulation time. Additionally, developing targeted delivery systems by conjugating Au NPs with homing peptides or antibodies can enhance their selective accumulation at injury sites and improve uptake by macrophages. Implementing controlled release mechanisms, such as hydrogels or liposomes, can ensure a sustained presence of Au NPs, facilitating prolonged interactions with immune cells. Furthermore, employing immune camouflage techniques—such as coating Au NPs with natural cell membranes or biomimetic materials—can effectively evade immune recognition, reducing detection and clearance. Ultimately, these design strategies should prioritize enhancing the immunomodulatory effects of Au NPs, particularly in promoting desired macrophage polarization and muscle regeneration. By creating a favorable microenvironment for tissue repair over an extended period, these optimized nanoparticles can significantly contribute to the advancement of therapeutic approaches for muscle injuries and related diseases.

(ii) Development of Targeted Delivery Systems

Effective delivery of Au NPs to the site of muscle injury is crucial for their therapeutic application. Untargeted delivery can lead to off-target effects, reducing efficacy and potentially causing adverse reactions. The development of targeted delivery systems, such as nanoparticles conjugated with homing peptides or antibodies, can significantly improve the selective accumulation of Au NPs at the injury site. These targeted systems can also enhance uptake by specific cell populations, such as macrophages, thereby increasing the local concentration of Au NPs and amplifying their immunomodulatory effects. Utilizing techniques such as receptor-mediated endocytosis can further enhance the specificity of Au NP delivery. By designing nanoparticles that mimic natural ligands for macrophage surface receptors, researchers can facilitate a more efficient uptake of Au NPs by these immune cells. This targeted approach not only maximizes the therapeutic potential of Au NPs but also minimizes systemic exposure, thereby reducing the risk of side effects.

(iii) Elucidation of Long-Term Effects

While Au NPs have shown promise in preclinical studies, the long-term effects of their use on muscle tissue and the immune system remain inadequately understood. Chronic exposure to Au NPs could potentially lead to unforeseen complications, such as sustained inflammation, fibrosis, or altered immune responses. Long-term studies are needed to monitor the persistence of Au NPs in tissues, their impact on tissue homeostasis, and any potential to induce adverse reactions. Understanding these long-term effects is essential for ensuring the safety and efficacy of Au NPs in clinical settings. This involves not only tracking the biodistribution and clearance of Au NPs over extended periods but also assessing their impact on cellular processes such as apoptosis, proliferation, and differentiation in muscle and immune cells. Establishing a comprehensive safety profile will be vital for gaining regulatory approval and fostering confidence in the clinical application of Au NP-based therapies.

As research in this field progresses, several future directions can be identified.

(i) Multifunctional Au NPs

Developing Au NPs with multiple functionalities, such as combined imaging and therapeutic capabilities, could significantly enhance their utility in regenerative medicine. This might involve conjugating Au NPs with both imaging agents and therapeutic drugs to allow for real-time monitoring of therapeutic effects while simultaneously delivering treatment. For instance, incorporating fluorescent or magnetic resonance imaging agents can help visualize the distribution and accumulation of Au NPs in vivo, providing valuable insights into their therapeutic efficacy and biodistribution patterns. Moreover, multifunctional Au NPs could enable the simultaneous delivery of multiple therapeutic agents, enhancing the overall effectiveness of treatment strategies. This could be particularly beneficial in complex scenarios where muscle injuries are accompanied by other pathological conditions, necessitating a multifaceted therapeutic approach.

(ii) Understanding Mechanisms of Action

Further research is needed to elucidate the precise mechanisms by which Au NPs modulate macrophage polarization and influence muscle regeneration. This includes understanding how Au NPs interact with cellular receptors and signaling pathways to affect macrophage behavior. The integration of bioinformatics and multi-omics analysis, along with machine learning algorithms, can provide deeper insights into the complex interactions between Au NPs and biological systems. These advanced technologies can help identify specific molecular pathways and biomarkers of successful macrophage polarization and muscle regeneration, facilitating the development of targeted therapies tailored to individual patient needs [196]. For instance, single-cell RNA sequencing (scRNA-seq) can be used to analyze the gene expression profiles of macrophages treated with Au NPs, revealing the specific pathways involved in their polarization. Spatial transcriptomics can provide insights into the spatial distribution of these macrophages within the muscle tissue, helping to understand the local effects of Au NPs. Additionally, machine learning algorithms can be employed to analyze large datasets generated from these techniques, identifying patterns and predicting outcomes that may not be apparent through traditional analysis methods. In summary, the future directions for research on Au NPs should include the application of cutting-edge technologies such as bioinformatics, multi-omics analysis, and machine learning. These tools will enhance our understanding of the mechanisms of action of Au NPs in modulating macrophage polarization and promoting skeletal muscle regeneration, ultimately leading to the development of more effective therapeutic strategies.

(iii) Clinical Translation

While Au NPs have demonstrated promise in preclinical studies, their long-term effects on muscle tissue and the immune system remain insufficiently understood. Chronic exposure to Au NPs may lead to unforeseen complications, including sustained inflammation, fibrosis, or altered immune responses. Therefore, long-term studies are essential to monitor the persistence of Au NPs in tissues, their impact on tissue homeostasis, and any potential adverse reactions. Understanding these long-term effects is crucial for ensuring the safety and efficacy of Au NPs in clinical settings. This includes tracking their biodistribution and clearance over extended periods, as well as assessing their influence on cellular processes such as apoptosis, proliferation, and differentiation in muscle and immune cells. Establishing a comprehensive safety profile will be vital for regulatory approval and instilling confidence in the clinical application of Au NP-based therapies. Recent clinical trials investigating Au NPs in other tissue repair contexts have yielded promising results, suggesting potential for their application in skeletal muscle regeneration. Translating these preclinical findings into clinical practice necessitates rigorous testing in human subjects to evaluate the safety and efficacy of Au NPs for treating muscle injuries and diseases. Clinical trials should focus on therapeutic outcomes, pharmacokinetics, biodistribution, and potential immunogenicity. Collaboration among researchers, clinicians, and regulatory bodies will be essential to establish clear guidelines for the clinical evaluation of Au NP-based therapies. This will help streamline the approval process and enhance the likelihood of successful integration into standard clinical practice.

(iv) Personalized Medicine

The development of personalized Au NP therapies based on individual patient characteristics could significantly improve treatment outcomes. This might involve tailoring the properties of Au NPs to match the specific needs of each patient, such as their unique immunological profiles or the specific characteristics of their muscle injuries. Personalized approaches could also extend to the timing and dosing of Au NP therapies, optimizing treatment regimens to maximize efficacy while minimizing side effects. Utilizing patient-derived cells to test the responsiveness of Au NPs in vitro could provide valuable insights into the most effective formulations for individual patients, paving the way for a more customized approach to muscle regeneration therapies.

(v) Combination Therapies

Exploring the potential of Au NPs in combination with other therapeutic modalities, such as stem cell therapy or physical therapy, could enhance their effectiveness in promoting muscle regeneration. For instance, combining Au NPs with stem cell therapies may create a synergistic effect, where Au NPs modulate the local immune environment to support stem cell survival, proliferation, and differentiation. Additionally, integrating Au NP treatments with physical rehabilitation strategies could optimize recovery outcomes, as enhanced muscle regeneration facilitated by Au NPs may work in tandem with physical therapy to restore muscle function more effectively. Investigating these combination therapies will require multidisciplinary approaches, bringing together expertise from materials science, immunology, and clinical rehabilitation.

In summary, addressing these challenges and exploring these future directions will be critical for unlocking the full potential of Au NPs in enhancing skeletal muscle regeneration. Continued research and collaboration across disciplines will pave the way for the development of safe, effective, and innovative Au NP-based therapies for muscle injuries and diseases.

## 7 Conclusion

Au NPs represent a significant innovation in the field of regenerative medicine, particularly for enhancing skeletal muscle regeneration. Their unique physicochemical properties enable them to effectively modulate macrophage polarization, presenting a novel therapeutic intervention strategy that harnesses the immune response to promote tissue repair and healing. A deeper understanding of the mechanisms by which Au NPs interact with macrophages is essential for optimizing their therapeutic potential. This includes elucidating the cellular and molecular pathways involved, the impact of Au NPs' physical and chemical properties on these interactions, and the subsequent effects on cytokine production, cell signaling, and tissue regeneration processes. Such insights will enable the development of more targeted and effective Au NP-based therapies. The design and engineering of Au NPs are critical factors influencing their effectiveness. Future research should focus on optimizing their size, shape, surface charge, and functionalization to maximize biocompatibility, cellular uptake, and immunomodulatory capabilities. Additionally, the development of multifunctional Au NPs that incorporate imaging or drug delivery capabilities may offer synergistic benefits in therapeutic applications. Before Au NPs can be successfully translated into clinical practice, thorough assessments of their safety and efficacy are necessary. This includes preclinical studies to evaluate potential toxicity, immune responses, and long-term effects on muscle tissue and the immune system. Understanding the biodistribution and clearance of Au NPs in vivo is crucial to anticipate and mitigate any adverse effects. Ultimately, with continued investigation and development, Au NPs may become a standard tool in treating muscle injuries and diseases, significantly improving patient outcomes and quality of life.

## CRediT authorship contribution statement

**Lining Xu:** Writing – original draft. **Jiahuang Qiu:** Writing – original draft. **Quanzhong Ren:** Writing – original draft. **Dingding Wang:** Writing – original draft. **Anyi Guo:** Writing – original draft. **Ling Wang:** Writing – original draft. **Kedong Hou:** Writing – original draft. **Renxian Wang:** Writing – review & editing. **Yajun Liu:** Writing – review & editing.

## Declaration of competing interest

The authors declare that they have no known competing financial interests or personal relationships that could have appeared to influence the work reported in this paper.

## Data Availability

No data was used for the research described in the article.

## References

[1] Hong X., Campanario S., Ramirez-Pardo I., Grima-Terren M., Isern J., Munoz-Canoves P. (2022). Stem cell aging in the skeletal muscle: the importance of communication. Ageing Res. Rev..

[2] Domingues-Faria C., Vasson M.P., Goncalves-Mendes N., Boirie Y., Walrand S. (2016). Skeletal muscle regeneration and impact of aging and nutrition. Ageing Res. Rev..

[3] Tidball J.G. (2017). Regulation of muscle growth and regeneration by the immune system. Nat. Rev. Immunol..

[4] Sousa N.S., Brás M.F., Antunes I.B., Lindholm P., Neves J., Sousa-Victor P. (2023). Aging disrupts MANF-mediated immune modulation during skeletal muscle regeneration. Nature aging.

[5] Chazaud B. (2020). Inflammation and skeletal muscle regeneration: leave it to the macrophages. Trends Immunol..

[6] Li T., Ma J., Wang W., Lei B. (2023). Bioactive MXene promoting angiogenesis and skeletal muscle regeneration through regulating M2 polarization and oxidation stress. Adv. Healthcare Mater..

[7] Zhang C., Cheng N., Qiao B., Zhang F., Wu J., Liu C., Li Y., Du J. (2020). Age-related decline of interferon-gamma responses in macrophage impairs satellite cell proliferation and regeneration. Journal of cachexia, sarcopenia and muscle.

[8] Bhattarai S., Li Q., Ding J., Liang F., Gusev E., Lapohos O., Fonseca G.J., Kaufmann E., Divangahi M., Petrof B.J. (2022). TLR4 is a regulator of trained immunity in a murine model of Duchenne muscular dystrophy. Nat. Commun..

[9] Shang M., Cappellesso F., Amorim R., Serneels J., Virga F., Eelen G., Carobbio S., Rincon M.Y., Maechler P., De Bock K., Ho P.C., Sandri M., Ghesquière B., Carmeliet P., Di Matteo M., Berardi E., Mazzone M. (2020). Macrophage-derived glutamine boosts satellite cells and muscle regeneration. Nature.

[10] Wang Y., Welc S.S., Wehling-Henricks M., Tidball J.G. (2018). Myeloid cell-derived tumor necrosis factor-alpha promotes sarcopenia and regulates muscle cell fusion with aging muscle fibers. Aging Cell.

[11] Acharyya S., Villalta S.A., Bakkar N., Bupha-Intr T., Janssen P.M., Carathers M., Li Z.W., Beg A.A., Ghosh S., Sahenk Z., Weinstein M., Gardner K.L., Rafael-Fortney J.A., Karin M., Tidball J.G., Baldwin A.S., Guttridge D.C. (2007). Interplay of IKK/NF-kappaB signaling in macrophages and myofibers promotes muscle degeneration in Duchenne muscular dystrophy. J. Clin. Investig..

[12] Southerland K.W., Xu Y., Peters D.T., Lin X., Wei X., Xiang Y., Fei K., Olivere L.A., Morowitz J.M., Otto J., Dai Q., Kontos C.D., Diao Y. (2023). Skeletal muscle regeneration failure in ischemic-damaged limbs is associated with pro-inflammatory macrophages and premature differentiation of satellite cells. Genome Med..

[13] Khlebtsov N., Dykman L. (2011). Biodistribution and toxicity of engineered gold nanoparticles: a review of in vitro and in vivo studies. Chem. Soc. Rev..

[14] Xu L., Xu M., Sun X., Feliu N., Feng L., Parak W.J., Liu S. (2023). Quantitative comparison of gold nanoparticle delivery via the enhanced permeation and retention (EPR) effect and mesenchymal stem cell (MSC)-Based targeting. ACS Nano.

[15] Del Pino P., Yang F., Pelaz B., Zhang Q., Kantner K., Hartmann R., Martinez de Baroja N., Gallego M., Möller M., Manshian B.B., Soenen S.J., Riedel R., Hampp N., Parak W.J. (2016). Basic physicochemical properties of polyethylene glycol coated gold nanoparticles that determine their interaction with cells. Angew. Chem..

[16] Ni C., Zhou J., Kong N., Bian T., Zhang Y., Huang X., Xiao Y., Yang W., Yan F. (2019). Gold nanoparticles modulate the crosstalk between macrophages and periodontal ligament cells for periodontitis treatment. Biomaterials.

[17] Raimondo T.M., Mooney D.J. (2018). Functional muscle recovery with nanoparticle-directed M2 macrophage polarization in mice. Proc. Natl. Acad. Sci. U. S. A.

[18] Corsi F., Carotenuto F., Di Nardo P., Teodori L. (2020). Harnessing inorganic nanoparticles to direct macrophage polarization for skeletal muscle regeneration. Nanomaterials.

[19] Relaix F., Bencze M., Borok M.J., Der Vartanian A., Gattazzo F., Mademtzoglou D., Perez-Diaz S., Prola A., Reyes-Fernandez P.C., Rotini A., Taglietti t. (2021). Perspectives on skeletal muscle stem cells. Nat. Commun..

[20] Jeong G.J., Castels H., Kang I., Aliya B., Jang Y.C. (2022). Nanomaterial for skeletal muscle regeneration. Tissue engineering and regenerative medicine.

[21] Brooks S.V., Guzman S.D., Ruiz L.P. (2023). Skeletal muscle structure, physiology, and function. Handb. Clin. Neurol..

[22] Sousa-Victor P., García-Prat L., Muñoz-Cánoves P. (2022). Control of satellite cell function in muscle regeneration and its disruption in ageing. Nat. Rev. Mol. Cell Biol..

[23] Murach K.A., Fry C.S., Dupont-Versteegden E.E., McCarthy J.J., Peterson C.A. (2021). Fusion and beyond: satellite cell contributions to loading-induced skeletal muscle adaptation. FASEB J..

[24] Yamakawa H., Kusumoto D., Hashimoto H., Yuasa S. (2020). Stem cell aging in skeletal muscle regeneration and disease. Int. J. Mol. Sci..

[25] Brett J.O., Arjona M., Ikeda M., Quarta M., de Morrée A., Egner I.M., Perandini L.A., Ishak H.D., Goshayeshi A., Benjamin D.I., Both P., Rodríguez-Mateo C., Betley M.J., Wyss-Coray T., Rando T.A. (2020). Exercise rejuvenates quiescent skeletal muscle stem cells in old mice through restoration of Cyclin D1. Nat. Metab..

[26] Chargé S.B., Rudnicki M.A. (2004). Cellular and molecular regulation of muscle regeneration. Physiol. Rev..

[27] Ratnayake D., Nguyen P.D., Rossello F.J., Wimmer V.C., Tan J.L., Galvis L.A., Julier Z., Wood A.J., Boudier T., Isiaku A.I., Berger S., Oorschot V., Sonntag C., Rogers K.L., Marcelle C., Lieschke G.J., Martino M.M., Bakkers J., Currie P.D. (2021). Macrophages provide a transient muscle stem cell niche via NAMPT secretion. Nature.

[28] Tidball J.G. (2011). Mechanisms of muscle injury, repair, and regeneration. Compr. Physiol..

[29] Tang Z., Zhou G., Xiao Y., Liu H., Chen X., Shen M. (2024). Allergic phenotypes and sarcopenia: evidence from observational studies and mendelian randomization analysis. Phenomics (Cham, Switzerland).

[30] Chi Z., Chen S., Yang D., Cui W., Lu Y., Wang Z., Li M., Yu W., Zhang J., Jiang Y., Sun R., Yu Q., Hu T., Lu X., Deng Q., Yang Y., Zhao T., Chang M., Li Y., Zhang X., Shang M., Xiao Q., Ding K., Wang D. (2024). Gasdermin D-mediated metabolic crosstalk promotes tissue repair. Nature.

[31] McNamara S.L., Seo B.R., Freedman B.R., Roloson E.B., Alvarez J.T., O'Neill C.T., Vandenburgh H.H., Walsh C.J., Mooney D.J. (2023). Anti-inflammatory therapy enables robot-actuated regeneration of aged muscle. Sci. Robot..

[32] Graca F.A., Stephan A., Minden-Birkenmaier B.A., Shirinifard A., Wang Y.D., Demontis F., Labelle M. (2023). Platelet-derived chemokines promote skeletal muscle regeneration by guiding neutrophil recruitment to injured muscles. Nat. Commun..

[33] Sun Z., Yang L., Kiram A., Yang J., Yang Z., Xiao L., Yin Y., Liu J., Mao Y., Zhou D., Yu H., Zhou Z., Xu D., Jia Y., Ding C., Guo Q., Wang H., Li Y., Wang L., Fu T., Hu S., Gan Z. (2023). FNIP1 abrogation promotes functional revascularization of ischemic skeletal muscle by driving macrophage recruitment. Nat. Commun..

[34] Uderhardt S., Martins A.J., Tsang J.S., Lämmermann T., Germain R.N. (2019). Resident macrophages cloak tissue microlesions to prevent neutrophil-driven inflammatory damage. Cell.

[35] Zhang J., Yang Y., Yang Z., Li T., Chen F. (2018). Snapshot: targeting macrophages as a candidate for tissue regeneration. Curr. Issues Mol. Biol..

[36] Eming S.A., Wynn T.A., Martin P. (2017). Inflammation and metabolism in tissue repair and regeneration. Science.

[37] Wynn T.A., Vannella K.M. (2016). Macrophages in tissue repair, regeneration, and fibrosis. Immunity.

[38] Mass E., Nimmerjahn F., Kierdorf K., Schlitzer A. (2023). Tissue-specific macrophages: how they develop and choreograph tissue biology. Nat. Rev. Immunol..

[39] Lazarov T., Juarez-Carreño S., Cox N., Geissmann F. (2023). Physiology and diseases of tissue-resident macrophages. Nature.

[40] Luo M., Zhao F., Cheng H., Su M., Wang Y. (2024). Macrophage polarization: an important role in inflammatory diseases. Front. Immunol..

[41] Chazaud B. (2020). A macrophage-derived adipokine supports skeletal muscle regeneration. Nat. Metab..

[42] Du H., Shih C.H., Wosczyna M.N., Mueller A.A., Cho J., Aggarwal A., Rando T.A., Feldman B.J. (2017). Macrophage-released ADAMTS1 promotes muscle stem cell activation. Nat. Commun..

[43] Varga T., Mounier R., Patsalos A., Gogolák P., Peloquin M., Horvath A., Pap A., Daniel B., Nagy G., Pintye E., Póliska S., Cuvellier S., Larbi S.B., Sansbury B.E., Spite M., Brown C.W., Chazaud B., Nagy L. (2016). Macrophage PPARγ, a lipid activated transcription factor controls the growth factor GDF3 and skeletal muscle regeneration. Immunity.

[44] Dort J., Fabre P., Molina T., Dumont N.A. (2019). Macrophages are key regulators of stem cells during skeletal muscle regeneration and diseases. Stem Cell. Int..

[45] He Y., Heng Y., Qin Z., Wei X., Wu Z., Qu J. (2023). Intravital microscopy of satellite cell dynamics and their interaction with myeloid cells during skeletal muscle regeneration. Sci. Adv..

[46] Tacke F. (2017). Targeting hepatic macrophages to treat liver diseases. J. Hepatol..

[47] Nagarsheth N., Wicha M.S., Zou W. (2017). Chemokines in the cancer microenvironment and their relevance in cancer immunotherapy. Nat. Rev. Immunol..

[48] Li X., Yao W., Yuan Y., Chen P., Li B., Li J., Chu R., Song H., Xie D., Jiang X., Wang H. (2017). Targeting of tumour-infiltrating macrophages via CCL2/CCR2 signalling as a therapeutic strategy against hepatocellular carcinoma. Gut.

[49] Bonapace L., Coissieux M.M., Wyckoff J., Mertz K.D., Varga Z., Junt T., Bentires-Alj M. (2014). Cessation of CCL2 inhibition accelerates breast cancer metastasis by promoting angiogenesis. Nature.

[50] Roelofs A.J., Thompson K., Gordon S., Rogers M.J. (2006). Molecular mechanisms of action of bisphosphonates: current status. Clin. Cancer Res. : an official journal of the American Association for Cancer Research.

[51] Xiao W., Liu Y., Chen P. (2016). Macrophage depletion impairs skeletal muscle regeneration: the roles of pro-fibrotic factors, inflammation, and oxidative stress. Inflammation.

[52] Liu X., Zeng Z., Zhao L., Chen P., Xiao W. (2019). Impaired skeletal muscle regeneration induced by macrophage depletion could Be partly ameliorated by MGF injection. Front. Physiol..

[53] Liu X., Liu Y., Zhao L., Zeng Z., Xiao W., Chen P. (2017). Macrophage depletion impairs skeletal muscle regeneration: the roles of regulatory factors for muscle regeneration. Cell Biol. Int..

[54] Li S., Wang Y., Wu M., Younis M.H., Olson A.P., Barnhart T.E., Engle J.W., Zhu X., Cai W. (2022). Spleen-targeted glabridin-loaded nanoparticles regulate polarization of monocyte/macrophage (M(o)/M(φ)) for the treatment of cerebral ischemia-reperfusion injury. Adv. Mater..

[55] Zhang J., Muri J., Fitzgerald G., Gorski T., Gianni-Barrera R., Masschelein E., D'Hulst G., Gilardoni P., Turiel G., Fan Z., Wang T., Planque M., Carmeliet P., Pellerin L., Wolfrum C., Fendt S.M., Banfi A., Stockmann C., Soro-Arnáiz I., Kopf M., De Bock K. (2020). Endothelial lactate controls muscle regeneration from ischemia by inducing M2-like macrophage polarization. Cell Metab..

[56] Tonkin J., Temmerman L., Sampson R.D., Gallego-Colon E., Barberi L., Bilbao D., Schneider M.D., Musarò A., Rosenthal N. (2015). Monocyte/Macrophage-derived IGF-1 orchestrates murine skeletal muscle regeneration and modulates autocrine polarization. Mol. Ther. : the journal of the American Society of Gene Therapy.

[57] Millozzi F., Papait A., Bouché M., Parolini O., Palacios D. (2023). Nano-immunomodulation: a new strategy for skeletal muscle diseases and aging?. Int. J. Mol. Sci..

[58] Chen W., Zhang F., Ju Y., Hong J., Ding Y. (2021). Gold nanomaterial engineering for macrophage-mediated inflammation and tumor treatment. Adv. Healthcare Mater..

[59] Xu L., Wang X., Wang R., Liu S., Xu M. (2023). Engineered macrophages: a safe-by-design approach for the tumor targeting delivery of sub-5 nm gold nanoparticles. Small.

[60] Xu L., Wang X., Xu M., Liu S. (2023). Single-particle hyperspectral imaging for monitoring of gold nanoparticle aggregates in macrophages. J. Phys. Chem. B.

[61] Jans H., Huo Q. (2012). Gold nanoparticle-enabled biological and chemical detection and analysis. Chem. Soc. Rev..

[62] Sharifi M., Attar F., Saboury A.A., Akhtari K., Hooshmand N., Hasan A., El-Sayed M.A., Falahati M. (2019). Plasmonic gold nanoparticles: optical manipulation, imaging, drug delivery and therapy. J. Contr. Release : official journal of the Controlled Release Society.

[63] Dreaden E.C., Alkilany A.M., Huang X., Murphy C.J., El-Sayed M.A. (2012). The golden age: gold nanoparticles for biomedicine. Chem. Soc. Rev..

[64] Oh N., Park J.H. (2014). Surface chemistry of gold nanoparticles mediates their exocytosis in macrophages. ACS Nano.

[65] Hajfathalian M., de Vries C.R., Hsu J.C., Amirshaghaghi A., Dong Y.C., Ren Z., Liu Y., Huang Y., Li Y., Knight S.A., Jonnalagadda P., Zlitni A., Grice E.A., Bollyky P.L., Koo H., Cormode D.P. (2023). Theranostic gold-in-gold cage nanoparticles enable photothermal ablation and photoacoustic imaging in biofilm-associated infection models. J. Clin. Investig..

[66] Niikura K., Matsunaga T., Suzuki T., Kobayashi S., Yamaguchi H., Orba Y., Kawaguchi A., Hasegawa H., Kajino K., Ninomiya T., Ijiro K., Sawa H. (2013). Gold nanoparticles as a vaccine platform: influence of size and shape on immunological responses in vitro and in vivo. ACS Nano.

[67] Huang L., Mao X., Li J., Li Q., Shen J., Liu M., Fan C., Tian Y. (2023). Nanoparticle spikes enhance cellular uptake via regulating myosin IIA recruitment. ACS Nano.

[68] Boisselier E., Astruc D. (2009). Gold nanoparticles in nanomedicine: preparations, imaging, diagnostics, therapies and toxicity. Chem. Soc. Rev..

[69] Zhou W., Gao X., Liu D., Chen X. (2015). Gold nanoparticles for in vitro diagnostics. Chem. Rev..

[70] Lu X., Punj D., Orrit M. (2022). Two-photon-excited single-molecule fluorescence enhanced by gold nanorod dimers. Nano Lett..

[71] Yadid M., Feiner R., Dvir T. (2019). Gold nanoparticle-integrated scaffolds for tissue engineering and regenerative medicine. Nano Lett..

[72] Goddard Z.R., Marín M.J., Russell D.A., Searcey M. (2020). Active targeting of gold nanoparticles as cancer therapeutics. Chem. Soc. Rev..

[73] Hastman D.A., Oh E., Melinger J.S., Green C.M., Thielemann A.J.P., Medintz I.L., Díaz S.A. (2024). Smaller gold nanoparticles release DNA more efficiently during fs laser pulsed optical heating. Small.

[74] Li N., Zhao P., Astruc D. (2014). Anisotropic gold nanoparticles: synthesis, properties, applications, and toxicity. Angew. Chem..

[75] Ojea-Jiménez I., García-Fernández L., Lorenzo J., Puntes V.F. (2012). Facile preparation of cationic gold nanoparticle-bioconjugates for cell penetration and nuclear targeting. ACS Nano.

[76] Kus-Liśkiewicz M., Fickers P., Ben Tahar I. (2021). Biocompatibility and cytotoxicity of gold nanoparticles: recent advances in methodologies and regulations. Int. J. Mol. Sci..

[77] Xu L., Wang X., Wang R., Liu S., Xu M. (2023). Engineered macrophages: a safe-by-design approach for the tumor targeting delivery of sub-5 nm gold nanoparticles. Small.

[78] Lu J., Gao X., Wang S., He Y., Ma X., Zhang T., Liu X. (2023). Advanced strategies to evade the mononuclear phagocyte system clearance of nanomaterials. Explorations.

[79] Raimondo T.M., Mooney D.J. (2021). Anti-inflammatory nanoparticles significantly improve muscle function in a murine model of advanced muscular dystrophy. Sci. Adv..

[80] Chan C.K.W., Szeto C.C., Lee L.K.C., Xiao Y., Yin B., Ding X., Lee T.W.Y., Lau J.Y.W., Choi C.H.J. (2023). A sub-10-nm, folic acid-conjugated gold nanoparticle as self-therapeutic treatment of tubulointerstitial fibrosis. Proc. Natl. Acad. Sci. U. S. A..

[81] Balasubramanian S.K., Jittiwat J., Manikandan J., Ong C.N., Yu L.E., Ong W.Y. (2010). Biodistribution of gold nanoparticles and gene expression changes in the liver and spleen after intravenous administration in rats. Biomaterials.

[82] Zhang X.D., Wu D., Shen X., Liu P.X., Fan F.Y., Fan S.J. (2012). In vivo renal clearance, biodistribution, toxicity of gold nanoclusters. Biomaterials.

[83] Elci S.G., Jiang Y., Yan B., Kim S.T., Saha K., Moyano D.F., Yesilbag Tonga G., Jackson L.C., Rotello V.M., Vachet R.W. (2016). Surface charge controls the suborgan biodistributions of gold nanoparticles. ACS Nano.

[84] Falagan-Lotsch P., Grzincic E.M., Murphy C.J. (2016). One low-dose exposure of gold nanoparticles induces long-term changes in human cells. Proc. Natl. Acad. Sci. U. S. A..

[85] Balfourier A., Kolosnjaj-Tabi J., Luciani N., Carn F., Gazeau F. (2020). Gold-based therapy: from past to present. Proc. Natl. Acad. Sci. U. S. A..

[86] Niżnik Ł., Noga M., Kobylarz D., Frydrych A., Krośniak A., Kapka-Skrzypczak L., Jurowski K. (2024). Gold nanoparticles (AuNPs)-Toxicity, safety and green synthesis: a critical review. Int. J. Mol. Sci..

[87] Chhour P., Naha P.C., O'Neill S.M., Litt H.I., Reilly M.P., Ferrari V.A., Cormode D.P. (2016). Labeling monocytes with gold nanoparticles to track their recruitment in atherosclerosis with computed tomography. Biomaterials.

[88] Yu C., Chen Z., Li X., Bao H., Wang Y., Zhang B., Huang J., Zhang Z. (2021). pH-triggered aggregation of gold nanoparticles for enhanced labeling and long-term CT imaging tracking of stem cells in pulmonary fibrosis treatment. Small.

[89] Bao H., Cheng S., Li X., Li Y., Yu C., Huang J., Zhang Z. (2022). Functional Au nanoparticles for engineering and long-term CT imaging tracking of mesenchymal stem cells in idiopathic pulmonary fibrosis treatment. Biomaterials.

[90] Tang S., Peng C., Xu J., Du B., Wang Q., Vinluan R.D., Yu M., Kim M.J., Zheng J. (2016). Tailoring renal clearance and tumor targeting of ultrasmall metal nanoparticles with particle density. Angew. Chem..

[91] Bourquin J., Milosevic A., Hauser D., Lehner R., Blank F., Petri-Fink A., Rothen-Rutishauser, Biodistribution B. (2018). Clearance, and long-term fate of clinically relevant nanomaterials. Adv. Mater..

[92] Yang X., Yang M., Pang B., Vara M., Xia Y. (2015). Gold nanomaterials at work in biomedicine. Chem. Rev..

[93] Li X., Wang B., Zhou S., Chen W., Chen H., Liang S., Zheng L., Yu H., Chu R., Wang M., Chai Z., Feng W. (2020). Surface chemistry governs the sub-organ transfer, clearance and toxicity of functional gold nanoparticles in the liver and kidney. J. Nanobiotechnol..

[94] Xie L., Zhang X., Chu C., Dong Y., Zhang T., Li X., Liu G., Cai W., Han S. (2021). Preparation, toxicity reduction and radiation therapy application of gold nanorods. J. Nanobiotechnol..

[95] Yousof S.M., Erfan H., Shehata S.A., Hosny M.M., El-Sayed K. (2023). Assessment of the potential cerebellar toxicity of gold nanoparticles on the structure and function of adult male albino rats. Biosci. Rep..

[96] Wang W., Wang J., Ding Y. (2020). Gold nanoparticle-conjugated nanomedicine: design, construction, and structure-efficacy relationship studies. J. Mater. Chem. B.

[97] Wang L., Rao Y., Liu X., Sun L., Gong J., Zhang H., Shen L., Bao A., Yang H. (2021). Administration route governs the therapeutic efficacy, biodistribution and macrophage targeting of anti-inflammatory nanoparticles in the lung. J. Nanobiotechnol..

[98] Joffe A.M., Bakalar M.H., Fletcher D.A. (2020). Macrophage phagocytosis assay with reconstituted target particles. Nat. Protoc..

[99] Nally F.K., De Santi C., McCoy C.E. (2019). Nanomodulation of macrophages in multiple sclerosis. Cells.

[100] Arami H., Khandhar A., Liggitt D., Krishnan K.M. (2015). In vivo delivery, pharmacokinetics, biodistribution and toxicity of iron oxide nanoparticles. Chem. Soc. Rev..

[101] Almeida J.P., Chen A.L., Foster A., Drezek R. (2011). In vivo biodistribution of nanoparticles. Nanomedicine.

[102] Salathia S., Gigliobianco M.R., Casadidio C., Di Martino P., Censi R. (2023). Hyaluronic acid-based nanosystems for CD44 mediated anti-inflammatory and antinociceptive activity. Int. J. Mol. Sci..

[103] Rios de la Rosa J.M., Tirella A., Gennari A., Stratford I.J., Tirelli N. (2017). The CD44-mediated uptake of hyaluronic acid-based carriers in macrophages. Adv. Healthcare Mater..

[104] Hlaing S.P., Cao J., Lee J., Kim J., Saparbayeva A., Kwak D., Kim H., Hwang S., Yun H., Moon H.R., Jung Y., Yoo J.W. (2022). Hyaluronic acid-conjugated PLGA nanoparticles alleviate ulcerative colitis via CD44-mediated dual targeting to inflamed colitis tissue and macrophages. Pharmaceutics.

[105] Thepen T., van Vuuren A.J., Kiekens R.C., Damen C.A., Vooijs W.C., van De Winkel J.G. (2000). Resolution of cutaneous inflammation after local elimination of macrophages. Nat. Biotechnol..

[106] Hristodorov D., Mladenov R., von Felbert V., Huhn M., Fischer R., Barth S., Thepen T. (2015). Targeting CD64 mediates elimination of M1 but not M2 macrophages in vitro and in cutaneous inflammation in mice and patient biopsies. mAbs.

[107] Yong S.B., Kim H.J., Kim J.K., Chung J.Y., Kim Y.H. (2017). Human CD64-targeted non-viral siRNA delivery system for blood monocyte gene modulation. Sci. Rep..

[108] Chia Z.C., Yang L.X., Cheng T.Y., Chen Y.J., Cheng H.L., Hsu F.T., Wang Y.J., Chen Y.Y., Huang T.C., Fang Y.S., Huang C.C. (2021). In situ formation of Au-glycopolymer nanoparticles for surface-enhanced Raman scattering-based biosensing and single-cell immunity. ACS Appl. Mater. Interfaces.

[109] Ali H.R., Selim S.A., Aili D. (2021). Effects of macrophage polarization on gold nanoparticle-assisted plasmonic photothermal therapy. RSC Adv..

[110] Fitzgerald K.A., Kagan J.C. (2020). Toll-like receptors and the control of immunity. Cell.

[111] Gao W., Wang L., Wang K., Sun L., Rao Y., Ma A., Zhang M., Li Q., Yang H. (2019). Enhanced anti-inflammatory activity of peptide-gold nanoparticle hybrids upon cigarette smoke extract modification through TLR inhibition and autophagy induction. ACS Appl. Mater. Interfaces.

[112] Rodriguez Lavado J., Sestito S.E., Cighetti R., Aguilar Moncayo E.M., Oblak A., Lainšček D., Jiménez Blanco J.L., García Fernández J.M., Ortiz Mellet C., Jerala R., Calabrese V., Peri F. (2014). Trehalose- and glucose-derived glycoamphiphiles: small-molecule and nanoparticle Toll-like receptor 4 (TLR4) modulators. J. Med. Chem..

[113] Dagvadorj J., Shimada K., Chen S., Jones H.D., Tumurkhuu G., Zhang W., Wawrowsky K.A., Crother T.R., Arditi M. (2015). Lipopolysaccharide induces alveolar macrophage necrosis via CD14 and the P2X7 receptor leading to interleukin-1α release. Immunity.

[114] Yang H., Kozicky L., Saferali A., Fung S.Y., Afacan N., Cai B., Falsafi R., Gill E., Liu M., Kollmann T.R., Hancock R.E., Sly L.M., Turvey S.E. (2016). Endosomal pH modulation by peptide-gold nanoparticle hybrids enables potent anti-inflammatory activity in phagocytic immune cells. Biomaterials.

[115] Kesharwani P., Ma R., Sang L., Fatima M., Sheikh A., Abourehab M.A.S., Gupta N., Chen Z.S., Zhou Y. (2023). Gold nanoparticles and gold nanorods in the landscape of cancer therapy. Mol. Cancer.

[116] Millozzi F., Milán-Rois P., Sett A., Delli Carpini G., De Bardi M., Gisbert-Garzarán M., Sandonà M., Rodríguez-Díaz C., Martínez-Mingo M., Pardo I., Esposito F., Viscomi M.T., Bouché M., Parolini O., Saccone V., Toulmé J.J., Somoza Á., Palacios D. (2025). Aptamer-conjugated gold nanoparticles enable oligonucleotide delivery into muscle stem cells to promote regeneration of dystrophic muscles. Nat. Commun..

[117] Jin S., Choi H., Seong D., You C.L., Kang J.S., Rho S., Lee W.B., Son D., Shin M. (2023). Injectable tissue prosthesis for instantaneous closed-loop rehabilitation. Nature.

[118] Sridhar S., Venugopal J.R., Sridhar R., Ramakrishna S. (2015). Cardiogenic differentiation of mesenchymal stem cells with gold nanoparticle loaded functionalized nanofibers. Colloids Surf. B Biointerfaces.

[119] Yang H.S., Lee B., Tsui J.H., Macadangdang J., Jang S.Y., Im S.G., Kim D.H. (2016). Electroconductive nanopatterned substrates for enhanced myogenic differentiation and maturation. Adv. Healthcare Mater..

[120] Peilin W., Ying P., Renyuan W., Zhuoxuan L., Zhenwu Y., Mai Z., Jianguo S., Hao Z., Gang Y., Lin L., Haodong L. (2023). Size-dependent gold nanoparticles induce macrophage M2 polarization and promote intracellular clearance of Staphylococcus aureus to alleviate tissue infection. Mater Today Bio.

[121] Gao W., Wang Y., Xiong Y., Sun L., Wang L., Wang K., Lu H.Y., Bao A., Turvey S.E., Li Q., Yang H. (2019). Size-dependent anti-inflammatory activity of a peptide-gold nanoparticle hybrid in vitro and in a mouse model of acute lung injury. Acta Biomater..

[122] Zhang S., Xie F., Li K., Zhang H., Yin Y., Yu Y., Lu G., Zhang S., Wei Y., Xu K., Wu Y., Jin H., Xiao L., Bao L., Xu C., Li Y., Lu Y., Gao J. (2022). Gold nanoparticle-directed autophagy intervention for antitumor immunotherapy via inhibiting tumor-associated macrophage M2 polarization. Acta Pharm. Sin. B.

[123] Su W.P., Chang L.C., Song W.H., Yang L.X., Wang L.C., Chia Z.C., Chin Y.C., Shan Y.S., Huang C.C., Yeh C.S. (2022). Polyaniline-based glyco-condensation on Au nanoparticles enhances immunotherapy in lung cancer. ACS Appl. Mater. Interfaces.

[124] Cheng J., Zhang Q., Fan S., Zhang A., Liu B., Hong Y., Guo J., Cui D., Song J. (2019). The vacuolization of macrophages induced by large amounts of inorganic nanoparticle uptake to enhance the immune response. Nanoscale.

[125] Deng Z.J., Liang M., Monteiro M., Toth I., Minchin R.F. (2011). Nanoparticle-induced unfolding of fibrinogen promotes Mac-1 receptor activation and inflammation. Nat. Nanotechnol..

[126] Oh N., Kim Y., Kweon H.S., Oh W.Y., Park J.H. (2018). Macrophage-mediated exocytosis of elongated nanoparticles improves hepatic excretion and cancer phototherapy. ACS Appl. Mater. Interfaces.

[127] Kang H., Wong S.H.D., Pan Q., Li G., Bian L. (2019). Anisotropic ligand nanogeometry modulates the adhesion and polarization state of macrophages. Nano Lett..

[128] Xia W., Song H.M., Wei Q., Wei A. (2012). Differential response of macrophages to core-shell Fe3O4@Au nanoparticles and nanostars. Nanoscale.

[129] Bhoge P.R., Mardhekar S., Toraskar S., Subramani B., Kikkeri R. (2022). Pairing nanoparticles geometry with TLR agonists to modulate immune responses for vaccine development. ACS Appl. Bio Mater..

[130] Mulens-Arias V., Nicolás-Boluda A., Carn F., Gazeau F. (2022). Cationic polyethyleneimine (PEI)-Gold nanocomposites modulate macrophage activation and reprogram mouse breast triple-negative MET-1 tumor immunological microenvironment. Pharmaceutics.

[131] Chen X., Gao C. (2017). Influences of size and surface coating of gold nanoparticles on inflammatory activation of macrophages. Colloids Surf. B Biointerfaces.

[132] Bai X., Chen D., Dai Y., Liang S., Song B., Guo J., Dai B., Zhang D., Feng L. (2021). Bone formation recovery with gold nanoparticle-induced M2 macrophage polarization in mice. Nanomedicine.

[133] Cao Y., Ding S., Zeng L., Miao J., Wang K., Chen G., Li C., Zhou J., Bian X.W., Tian G. (2021). Reeducating tumor-associated macrophages using CpG@Au nanocomposites to modulate immunosuppressive microenvironment for improved radio-immunotherapy. ACS Appl. Mater. Interfaces.

[134] Pallares R.M., Choo P., Cole L.E., Mirkin C.A., Lee A., Odom T.W. (2019). Manipulating immune activation of macrophages by tuning the oligonucleotide composition of gold nanoparticles. Bioconjug. Chem..

[135] Kim Y., Koo T.M., Thangam R., Kim M.S., Jang W.Y., Kang N., Min S., Kim S.Y., Yang L., Hong H., Jung H.J., Koh E.K., Patel K.D., Lee S., Fu H.E., Jeon Y.S., Park B.C., Kim S.Y., Park S., Lee J., Gu L., Kim D.H., Kim T.H., Lee K.B., Jeong W.K., Paulmurugan R., Kim Y.K., Kang H. (2022). Submolecular ligand size and spacing for cell adhesion. Adv. Mater..

[136] Bastús N.G., Sánchez-Tilló E., Pujals S., Farrera C., López C., Giralt E., Celada A., Lloberas J., Puntes V. (2009). Homogeneous conjugation of peptides onto gold nanoparticles enhances macrophage response. ACS Nano.

[137] Hung H.S., Yang Y.C., Chang C.H., Chang K.B., Shen C.C., Tang C.L., Liu S.Y., Lee C.H., Yen C.M., Yang M.Y. (2022). Neural differentiation potential of mesenchymal stem cells enhanced by biocompatible chitosan-gold nanocomposites. Cells.

[138] Cai R., Ren J., Ji Y., Wang Y., Liu Y., Chen Z., Farhadi Sabet Z., Wu X., Lynch I., Chen C. (2020). Corona of thorns: the surface chemistry-mediated protein corona perturbs the recognition and immune response of macrophages. ACS Appl. Mater. Interfaces.

[139] Ma Y., Hong J., Ding Y. (2020). Biological behavior regulation of gold nanoparticles via the protein corona. Adv. Healthcare Mater..

[140] Xu L., Xu M., Wang R., Yin Y., Lynch I., Liu S. (2020). The crucial role of environmental coronas in determining the biological effects of engineered nanomaterials. Small.

[141] Yang H., Lu S., Wang S., Liu L., Zhu B., Yu S., Yang S., Chang J. (2021). Evolution of the protein corona affects macrophage polarization. Int. J. Biol. Macromol..

[142] Wang L., Zhang H., Sun L., Gao W., Xiong Y., Ma A., Liu X., Shen L., Li Q., Yang H. (2020). Manipulation of macrophage polarization by peptide-coated gold nanoparticles and its protective effects on acute lung injury. J. Nanobiotechnol..

[143] Liao W., Ni C., Ge R., Li Y., Jiang S., Yang W., Yan F. (2024). Nel-like molecule type 1 combined with gold nanoparticles modulates macrophage polarization, osteoclastogenesis, and oral microbiota in periodontitis. ACS Appl. Mater. Interfaces.

[144] Aili M., Zhou K., Zhan J., Zheng H., Luo F. (2023). Anti-inflammatory role of gold nanoparticles in the prevention and treatment of Alzheimer's disease. J. Mater. Chem. B.

[145] Hung H.S., Kung M.L., Chen F.C., Ke Y.C., Shen C.C., Yang Y.C., Tang C.M., Yeh C.A., Hsieh H.H., Hsu S.H. (2021). Nanogold-carried graphene oxide: anti-inflammation and increased differentiation capacity of mesenchymal stem cells. Nanomaterials.

[146] Guan C.M., Chinen A.B., Ferrer J.R., Ko C.H., Mirkin C.A. (2019). Impact of sequence specificity of spherical nucleic acids on macrophage activation in vitro and in vivo. Mol. Pharm..

[147] Nelms K., Keegan A.D., Zamorano J., Ryan J.J., Paul W.E. (1999). The IL-4 receptor: signaling mechanisms and biologic functions. Annu. Rev. Immunol..

[148] LaPorte S.L., Juo Z.S., Vaclavikova J., Colf L.A., Qi X., Heller N.M., Keegan A.D., Garcia K.C. (2008). Molecular and structural basis of cytokine receptor pleiotropy in the interleukin-4/13 system. Cell.

[149] Gunassekaran G.R., Poongkavithai Vadevoo S.M., Baek M.C., Lee B. (2021). M1 macrophage exosomes engineered to foster M1 polarization and target the IL-4 receptor inhibit tumor growth by reprogramming tumor-associated macrophages into M1-like macrophages. Biomaterials.

[150] Sun L., Liu Y., Liu X., Wang R., Gong J., Saferali A., Gao W., Ma A., Ma H., Turvey S.E., Fung S.Y., Yang H. (2022). Nano-enabled reposition of proton pump inhibitors for TLR inhibition: toward A new targeted nanotherapy for acute lung injury. Adv. Sci..

[151] Zhang X., Liu Y., Jiang M., Mas-Rosario J.A., Fedeli S., Cao-Milan R., Liu L., Winters K.J., Hirschbiegel C.M., Nabawy A., Huang R., Farkas M.E., Rotello V.M. (2024). Polarization of macrophages to an anti-cancer phenotype through in situ uncaging of a TLR 7/8 agonist using bioorthogonal nanozymes. Chem. Sci..

[152] Sun L., Wang R., Wu C., Gong J., Ma H., Fung S.Y., Yang H. (2021). The modulatory activity of tryptophan displaying nanodevices on macrophage activation for preventing acute lung injury. Front. Immunol..

[153] Wu Y., Deshpande A., Geraci N., Budde P., Sellers V., Velisetty P., Sun C.C., Strand F., Bhavsar C., Niewold T.B., Jensen M.A., Kalatskaya I., Sarin K.Y., Fiorentino D., Bender A.T. (2025). TLR7/8 activation in immune cells and muscle by RNA-containing immune complexes: role in inflammation and the pathogenesis of myositis. Arthritis Rheumatol..

[154] Bastús N.G., Sánchez-Tilló E., Pujals S., Farrera C., Kogan M.J., Giralt E., Celada A., Lloberas J., Puntes V. (2009). Peptides conjugated to gold nanoparticles induce macrophage activation. Mol. Immunol..

[155] Duncan J.B.W., Basu S., Vivekanand P. (2023). Honey gold nanoparticles attenuate the secretion of IL-6 by LPS-activated macrophages. PLoS One.

[156] Rizwan H., Mohanta J., Si S., Pal A. (2017). Gold nanoparticles reduce high glucose-induced oxidative-nitrosative stress regulated inflammation and apoptosis via tuberin-mTOR/NF-κB pathways in macrophages. Int. J. Nanomed..

[157] Wang S., Lu M., Wang W., Yu S., Yu R., Cai C., Li Y., Shi Z., Zou J., He M., Xie W., Yu D., Jin H., Li H., Xiao W., Fan C., Wu F., Li Y., Liu S. (2022). Macrophage polarization modulated by NF-κB in polylactide membranes-treated peritendinous adhesion. Small.

[158] Ma J.S., Kim W.J., Kim J.J., Kim T.J., Ye S.K., Song M.D., Kang H., Kim D.W., Moon W.K., Lee K.H. (2010). Gold nanoparticles attenuate LPS-induced NO production through the inhibition of NF-kappaB and IFN-beta/STAT1 pathways in RAW264.7 cells. Nitric Oxide : biology and chemistry.

[159] Liu C., Hu F., Jiao G., Guo Y., Zhou P., Zhang Y., Zhang Z., Yi J., You Y., Li Z., Wang H., Zhang X. (2022). Dental pulp stem cell-derived exosomes suppress M1 macrophage polarization through the ROS-MAPK-NFκB P65 signaling pathway after spinal cord injury. J. Nanobiotechnol..

[160] Han L., Haefner V., Yu Y., Han B., Ren H., Irmler M., Beckers J., Liu Q., Feuchtinger A., Yildirim A.O., Adler H., Stoeger T. (2023). Nanoparticle-exposure-triggered virus reactivation induces lung emphysema in mice. ACS Nano.

[161] Hu W.M., Liu S.Q., Zhu K.F., Li W., Yang Z.J., Yang Q., Zhu Z.C., Chang J. (2023). The ALOX5 inhibitor Zileuton regulates tumor-associated macrophage M2 polarization by JAK/STAT and inhibits pancreatic cancer invasion and metastasis. Int. Immunopharmacol..

[162] Fortelny N., Farlik M., Fife V., Gorki A.D., Lassnig C., Maurer B., Meissl K., Dolezal M., Boccuni L., Ravi Sundar Jose Geetha A., Akagha M.J., Karjalainen A., Shoebridge S., Farhat A., Mann U., Jain R., Tikoo S., Zila N., Esser-Skala W., Krausgruber T., Sitnik K., Penz T., Hladik A., Suske T., Zahalka S., Senekowitsch M., Barreca D., Halbritter F., Macho-Maschler S., Weninger W., Neubauer H.A., Moriggl R., Knapp S., Sexl V., Strobl B., Decker T., Müller M., Bock C. (2024). JAK-STAT signaling maintains homeostasis in T cells and macrophages. Nat. Immunol..

[163] Yang X., Wang Q., Shao F., Zhuang Z., Wei Y., Zhang Y., Zhang L., Ren C., Wang H. (2024). Cell volume regulation modulates macrophage-related inflammatory responses via JAK/STAT signaling pathways. Acta Biomater..

[164] Lu C., Xue L., Luo K., Liu Y., Lai J., Yao X., Xue Y., Huo W., Meng C., Xia D., Gao X., Yuan Q., Cao K. (2023). Colon-accumulated gold nanoclusters alleviate intestinal inflammation and prevent secondary colorectal carcinogenesis via Nrf2-dependent macrophage reprogramming. ACS Nano.

[165] Luo Y.H., Cheng H.J., Tsai F.Y., Tsou T.C., Lin S.Y., Lin P. (2020). Primary amine modified gold nanodots regulate macrophage function and antioxidant response: potential therapeutics targeting of Nrf2. Int. J. Nanomed..

[166] Kim H.S., Lee S., Lee D.Y. (2023). Aurozyme: a revolutionary nanozyme in colitis, switching peroxidase-like to catalase-like activity. Small.

[167] Nguyen H.T., Shen H. (2016). The effect of PEGylation on the stimulation of IL-1β by gold (Au) nanoshell/silica core nanoparticles. J. Mater. Chem. B.

[168] Sun L., Liu Y., Yang N., Ye X., Liu Z., Wu J., Zhou M., Zhong W., Cao M., Zhang J., Mequanint K., Xing M., Liao W. (2023). Gold nanoparticles inhibit tumor growth via targeting the Warburg effect in a c-Myc-dependent way. Acta Biomater..

[169] Park J.Y., Kwon S., Kim S.H., Kang Y.J., Khang D. (2020). Triamcinolone-gold nanoparticles repolarize synoviocytes and macrophages in an inflamed synovium. ACS Appl. Mater. Interfaces.

[170] Pinho R.A., Haupenthal D.P.S., Fauser P.E., Thirupathi A., Silveira P.C.L. (2022). Gold nanoparticle-based therapy for muscle inflammation and oxidative stress. J. Inflamm. Res..

[171] Liu P.S., Wang H., Li X., Chao T., Teav T., Christen S., Di Conza G., Cheng W.C., Chou C.H., Vavakova M., Muret C., Debackere K., Mazzone M., Huang H.D., Fendt S.M., Ivanisevic J., Ho P.C. (2017). α-ketoglutarate orchestrates macrophage activation through metabolic and epigenetic reprogramming. Nat. Immunol..

[172] Yan J., Horng T. (2020). Lipid metabolism in regulation of macrophage functions. Trends Cell Biol..

[173] Li M., Yang Y., Xiong L., Jiang P., Wang J., Li C. (2023). Metabolism, metabolites, and macrophages in cancer. J. Hematol. Oncol..

[174] Dey A.K., Gonon A., Pécheur E.I., Pezet M., Villiers C., Marche P.N. (2021). Impact of gold nanoparticles on the functions of macrophages and dendritic cells. Cells.

[175] Li K., Lin C., Li M., Xu K., He Y., Mao Y., Lu L., Geng W., Li X., Luo Z., Cai K. (2022). Multienzyme-like reactivity cooperatively impairs glutathione peroxidase 4 and ferroptosis suppressor protein 1 pathways in triple-negative breast cancer for sensitized ferroptosis therapy. ACS Nano.

[176] Patel S., Yin P.T., Sugiyama H., Lee K.B. (2015). Inducing stem cell myogenesis using NanoScript. ACS Nano.

[177] Chen J., Ma Q., Li M., Chao D., Huang L., Wu W., Fang Y., Dong S. (2021). Glucose-oxidase like catalytic mechanism of noble metal nanozymes. Nat. Commun..

[178] Zhang D., Tang Z., Huang H., Zhou G., Cui C., Weng Y., Liu W., Kim S., Lee S., Perez-Neut M., Ding J., Czyz D., Hu R., Ye Z., He M., Zheng Y.G., Shuman H.A., Dai L., Ren B., Roeder R.G., Becker L., Zhao Y. (2019). Metabolic regulation of gene expression by histone lactylation. Nature.

[179] Liao Z., Liu T., Yao Z., Hu T., Ji X., Yao B. (2024). Harnessing Stimuli‐responsive Biomaterials for Advanced Biomedical Applications.

[180] Farr A.C., Hogan K.J., Mikos A.G. (2020). Nanomaterial additives for fabrication of stimuli-responsive skeletal muscle tissue engineering constructs. Adv. Healthcare Mater..

[181] Ramey-Ward A.N., Dong Y., Yang J., Ogasawara H., Bremer-Sai E.C., Brazhkina O., Franck C., Davis M., Salaita K. (2023). Optomechanically actuated hydrogel platform for cell stimulation with spatial and temporal resolution. ACS Biomater. Sci. Eng..

[182] Ramey-Ward A.N., Su H., Salaita K. (2020). Mechanical stimulation of adhesion receptors using light-responsive nanoparticle actuators enhances myogenesis. ACS Appl. Mater. Interfaces.

[183] Marino A., Arai S., Hou Y., Degl'Innocenti A., Cappello V., Mazzolai B., Chang Y.T., Mattoli V., Suzuki M., Ciofani G. (2017). Gold nanoshell-mediated remote myotube activation. ACS Nano.

[184] Ge J., Liu K., Niu W., Chen M., Wang M., Xue Y., Gao C., Ma P.X., Lei B. (2018). Gold and gold-silver alloy nanoparticles enhance the myogenic differentiation of myoblasts through p38 MAPK signaling pathway and promote in vivo skeletal muscle regeneration. Biomaterials.

[185] Darweesh R.S., Ayoub N.M., Nazzal S. (2019). Gold nanoparticles and angiogenesis: molecular mechanisms and biomedical applications. Int. J. Nanomed..

[186] Bartczak D., Muskens O.L., Sanchez-Elsner T., Kanaras A.G., Millar T.M. (2013). Manipulation of in vitro angiogenesis using peptide-coated gold nanoparticles. ACS Nano.

[187] Best T.M., Gharaibeh B., Huard J. (2013). Stem cells, angiogenesis and muscle healing: a potential role in massage therapies?. Br. J. Sports Med..

[188] Wei S.C., Chang L., Huang C.C., Chang H.T. (2019). Dual-functional gold nanoparticles with antimicrobial and proangiogenic activities improve the healing of multidrug-resistant bacteria-infected wounds in diabetic mice. Biomater. Sci..

[189] Kjaer M. (2004). Role of extracellular matrix in adaptation of tendon and skeletal muscle to mechanical loading. Physiol. Rev..

[190] Chang M., Nguyen T.T. (2021). Strategy for treatment of infected diabetic foot ulcers. Accounts Chem. Res..

[191] Wu Y.Z., Tsai Y.Y., Chang L.S., Chen Y.J. (2021). Evaluation of gallic acid-coated gold nanoparticles as an anti-aging ingredient. Pharmaceuticals.

[192] El Ketara S., Ford N.L. (2020). Time-course study of a gold nanoparticle contrast agent for cardiac-gated micro-CT imaging in mice. Biomedical physics & engineering express.

[193] Kee P.H., Danila D. (2018). CT imaging of myocardial scar burden with CNA35-conjugated gold nanoparticles. Nanomedicine.

[194] Bi D., Shi L., Li B., Li Y., Liu C., Le L.H., Luo J., Wang S., Ta D. (2024). The protocol of ultrasonic backscatter measurements of musculoskeletal properties. Phenomics (Cham, Switzerland).

[195] Jokerst J.V., Thangaraj M., Kempen P.J., Sinclair R., Gambhir S.S. (2012). Photoacoustic imaging of mesenchymal stem cells in living mice via silica-coated gold nanorods. ACS Nano.

[196] Tong L., Wijnen A.J.v., Wang H., Chen D. (2024). Advancing bone biology: the mutual promotion of biology and pioneering technologies. The Innovation Life.

